# Enhancing graduate students’ evaluative skills through AI-supported collaborative marking

**DOI:** 10.3389/fpsyg.2026.1739222

**Published:** 2026-03-31

**Authors:** Di Wu, Tiong-Thye Goh, Dexin Chen, Huan Yin

**Affiliations:** 1 Education College, Hubei University, Wuhan, China; 2 School of Information Management, Victoria University of Wellington, Wellington, New Zealand

**Keywords:** academic evaluation, AI agent, graduate education, human-AI collaboration, personalized intelligent academic guidance

## Abstract

Developing reliable evaluative judgment is a central and cognitively demanding task in graduate education, as assessing research writing requires integrating multiple quality criteria while regulating subjective bias. Effective evaluation relies on learners’ ability to internalize standards, monitor judgment accuracy, and calibrate decisions against expert benchmarks. Although prior AI-assisted assessment research has predominantly focused on optimizing predictive accuracy and scoring agreement with human raters, comparatively less attention has been paid to how AI-supported marking may function as a psychologically mediated process shaping learners’ evaluative judgment. Grounded in evaluative judgment theory and informed by self-regulated learning perspectives, this study conceptualizes AI-supported collaborative marking as a metacognitive scaffold that externalizes expert criteria, facilitates discrepancy detection, and supports reflective calibration. Using a mixed-method approach, 121 graduate research papers were assessed under four conditions: student-only marking, AI-only marking, retrieval-augmented AI marking, and AI-supported collaborative marking. Papers were evaluated across four dimensions including rigor, originality, significance, and academic conventions, with outcomes benchmarked against expert judgments. Results showed that AI-supported collaborative marking reduced deviations from expert ratings and improved inter-rater consistency compared with student-only and AI-only conditions, with MAE decreasing from 3.260 to 1.221. Behavioral sequence analyses revealed systematic differences in evaluative behaviors. Senior graduate students exhibited more developed metacognitive monitoring, reflective reasoning, and structured cycles of planning, monitoring, and reflection. In contrast, junior students relied more on exploratory and trial-and-error strategies, highlighting developmental differences in self-regulated evaluative competence. Overall, the findings indicate that AI-supported collaborative marking enhances assessment accuracy, and is associated with observable changes in students’ evaluative interaction patterns and reflective behaviors, which may contribute to the development of academic assessment literacy in graduate education.

## 1 Introduction

In graduate education, research paper assignment plays a crucial role in shaping students’ evaluative judgment, reflective reasoning, and long-term academic development (Cano García et al., 2024). Assessment is not merely a mechanism for assigning grades, but a cognitively demanding activity through which learners interpret criteria, monitor reasoning, and recalibrate judgments (Boud, 2020). Sustaining the quality and fairness of this process is increasingly challenging, especially in the era of artificial intelligence, which can both augment and complicate assessment practices (Xie, 2024). Effective higher education now requires not only accurate evaluation mechanisms but also learning environments that actively support students’ cognitive and metacognitive engagement with evaluation itself (Lu et al., 2021; Ajjawi et al., 2018). When marking is approached as a learning-oriented practice rather than a mere grading task, it can promote reflective engagement and long-term academic growth (Mcconlogue, 2020).

Recent advances in artificial intelligence have sparked interest not only in system performance but also in how AI can support students’ cognitive and metacognitive processes during assessment (Ouyang et al., 2022; Crompton and Burke, 2023). Advances in large language models (LLMs) now enable AI systems not only to assign marks but also to provide detailed rubric-based feedback. Yet, such systems still struggle with higher-order dimensions such as originality, quality of argumentation, and disciplinary nuance (Misgna et al., 2024), highlighting the limits of treating AI as an autonomous evaluator rather than as a learning support.

Studies have shown that machine-extracted insights can enhance the effectiveness of human markers and support collaboration in authentic instructional settings (Funayama et al., 2022; Li et al., 2025). In graduate education, this collaboration holds particular promise. Unlike undergraduates, graduate students are expected not only to master disciplinary content but also to develop familiarity with academic conventions, originality, and methodological rigor (Goodman et al., 2020). However, traditional supervision and peer review practices often provide limited guidance on how to apply assessment criteria consistently, constraining the development of evaluative judgment as a learnable competence (Taylor et al., 2024). AI-supported collaborative marking offers a structured environment in which students can practice assessment, receive immediate feedback, and progressively internalize expert standards. Recent studies have further shown that when AI is integrated into peer assessment, it can enhance both the consistency of marks and the quality of feedback, while providing students with rubric-aligned guidance that builds their confidence and fosters the internalization of expert criteria (Topping et al., 2025).

Rather than treating AI as a scoring substitute, this study conceptualizes AI-supported collaborative marking as a structured interaction designed to foster evaluative judgment development. This study adopts evaluative judgment theory as the primary theoretical lens. Within this perspective, high-quality judgment requires the internalization of standards and the capacity to compare one’s work with those standards. Self-regulated learning is introduced to explain the cyclical processes of monitoring and reflection through which evaluative standards are internalized. Cognitive load theory further clarifies how structured AI prompts reduce extraneous demands and support higher-order calibration.

The collaborative assessment process can be understood as an SRL-informed calibration cycle that involves planning, monitoring, and reflection. During evaluation tasks, learners must judge the quality of their decisions, compare them with external standards, and adjust their reasoning. These processes align closely with both self-regulated learning and evaluative judgment theory (Ibarra-Sáiz et al., 2025; Ortega-Ruipérez and Correa-Gorospe, 2024). In this cycle, AI-generated prompts, evidence checks, and structured feedback act as externalized metacognitive supports. They make expert criteria explicit and highlight discrepancies between student judgments and benchmark standards, which stimulates reflective monitoring and revision (Tomisu et al., 2025). And academic evaluation tasks impose high cognitive load because learners must consider multiple criteria and sources of evidence simultaneously. From a cognitive load perspective, structured AI interaction reduces extraneous demands by organizing evaluation steps and foregrounding key standards, allowing learners to focus on higher-order analysis and judgment calibration (Yao and Fan, 2025). Together, metacognitive regulation and optimized cognitive resource allocation provide a theoretical perspective for interpreting how AI-supported interaction may support students’ engagement with evaluative standards and reflective judgment processes.

Despite this theoretical potential, empirical research on how students cognitively engage with AI during assessment remains limited. There is a lack of evidence explaining how student–AI collaboration shapes evaluative reasoning processes, behavioral strategies, and judgment consistency (Topping et al., 2025). Importantly, this study differs from prior AI-assisted peer review and automated scoring research in three key respects. First, existing automated scoring studies primarily evaluate algorithmic accuracy or agreement with human raters, whereas this study examines how AI interaction reshapes students’ evaluative reasoning processes. Second, prior AI-assisted peer review research often focuses on feedback efficiency or revision quality, while the present study foregrounds the development of evaluative judgment as a learning outcome. Third, by embedding AI within a theoretically grounded calibration cycle, this study positions AI not as a predictive model but as a metacognitive partner that supports standards internalization and judgment refinement.

Accordingly, the present study poses the following research questions:

RQ1: How does AI-supported collaborative marking improve the accuracy and consistency of academic assessing?

RQ2: In what ways do AI-supported collaborative marking and AI without RAG model differ from expert assessing?

RQ3: What are the changing characteristics of graduate students' interaction strategies and behavioral patterns across different years of study during collaboration with AI agents?

To answer these questions, we adopted a mixed-methods approach to investigate AI-supported collaborative marking in graduate-level academic evaluation. The primary aim of the collaborative mechanism is not only to enhance scoring reliability, but to engage students in evaluative activity as a learning process, enabling them to compare judgments, reflect on discrepancies, and internalize expert standards. Through repeated interaction with AI-supported feedback, students are expected to develop stronger evaluative judgment, metacognitive awareness, and academic assessment literacy, thereby supporting both their writing development and long-term academic competence.

## 2 Literature review

### 2.1 Psychological foundations of evaluative judgment in AI-supported academic evaluation

Academic evaluation is fundamentally a psychological activity that requires learners to interpret quality criteria, monitor the adequacy of their judgments, and engage in reflective reasoning to recalibrate decisions. This process has been conceptualized through complementary theoretical lenses, including evaluative judgment theory, self-regulated learning, and cognitive load theory, which together explain how learners develop the capacity to assess academic work accurately and independently (Raković et al., 2022; de Bruin et al., 2020; Chong, 2021).

Prior research conceptualizes evaluative judgment as involving quality standards interpretation, reflective reasoning, and expert benchmark comparison (Bearman et al., 2024; Ibarra-Sáiz et al., 2025; Ortega-Ruipérez and Correa-Gorospe, 2024). Reflective reasoning enables learners to interrogate initial judgments and justify decisions, aligning them with established criteria. Self-regulated learning explains how this capacity develops through forethought, monitoring, and reflection cycles (Banihashem et al., 2025; Zimmerman and Schunk, 2011). From a cognitive load perspective, evaluation imposes substantial working memory demands; AI can scaffold this process by structuring steps and reducing extraneous load, freeing resources for higher-order analysis (Yao and Fan, 2025).

Yet these frameworks remain largely unintegrated in AI-supported assessment models. While AI increasingly supports metacognitive processes, over one-third of studies lack theoretical grounding, with SRL’s motivational dimension particularly underexplored (Banihashem et al., 2025). Engagement quality critically mediates outcomes: active interrogation strengthens regulation (Vo et al., 2024; Onan et al., 2024; Zheng et al., 2023), whereas excessive dependence undermines higher-order monitoring and evaluative accuracy (Romeo and Conti, 2025; Zhai et al., 2024). The OECD warns that overreliance may decrease metacognitive engagement, disconnecting performance from learning (OECD, 2026).

The core issue is not whether AI supports evaluation, but which conditions cultivate effective evaluative reasoning. Structured metacognitive requirements, such as explaining reasoning or rating confidence prior to AI feedback, may prove more impactful than feedback sophistication (Alsaiari et al., 2026). Yet current applications predominantly prioritize delivery accuracy over theorizing how AI reshapes evaluative reasoning, leaving underexplored a psychologically grounded calibration process coordinating cognitive load, metacognitive monitoring, and standards internalization.

Within the framework of the present study, AI functions as a regulatory resource that may help structure evaluative tasks and organize information during assessment activities. From a theoretical perspective, such structured interaction can be interpreted as potentially reducing extraneous cognitive demands and supporting reflective comparison with evaluation criteria. This conceptualization provides the psychological foundation for examining how student–AI collaborative marking shapes evaluative reasoning, behavioral regulation, and judgment consistency in graduate education.

### 2.2 Challenges of traditional human scoring and peer review

Traditional human-based marking faces well-documented challenges. Rater fatigue and cognitive overload from large-scale evaluation lead to score variability and delayed feedback (Mahshanian and Shahnazari, 2020; Ling et al., 2014; Ningrum and Crosthwaite, 2020). Human scoring is also prone to subjectivity and inconsistency in applying criteria, with variations in severity, leniency, and rubric interpretation across and within raters, compromising reliability and fairness (Barkaoui, 2011; Palermo, 2022). These delays undermine formative value, reducing student motivation and timely revision opportunities (Fisher et al., 2025). While instructor scoring ensures reliability under controlled conditions, it demands substantial resources, scales poorly, and struggles to provide sustained developmental feedback. Manual scoring alone yields delayed, limited feedback, constraining pedagogical utility, whereas automation addresses consistency and timeliness shortcomings (Han and Lu, 2021). Moreover, the high costs and labor-intensive nature of human grading raise concerns about its sustainability in large-scale applications, motivating the adoption of automated approaches that partially substitute or reduce human involvement (Morris et al., 2024; National Academies of Sciences, Engineering, and Medicine, 2022).

These challenges have prompted the exploration of complementary instructional approaches, such as peer review, which places students in the role of evaluators. Unlike instructor-only marking, peer review emphasizes learning through evaluating others’ work, fostering internalization of academic standards and evaluative judgment (Yin et al., 2022). Research indicates that peer evaluation strengthens critical thinking, reflective learning, and writing development by prompting analysis of others’ work and generating internal feedback for revision (Wei and Liu, 2024; Boillos, 2024; Chen and Cui, 2022; Alemdag and Narciss, 2025). Despite its instructional value, peer review is constrained by variations in feedback quality, inconsistent application of scoring criteria, and issues of fairness, particularly when participants have limited training (Zong et al., 2020; Lerchenfeldt et al., 2019; Anderson et al., 2020). While peer evaluation demonstrates developmental potential through evaluative practice, it remains vulnerable to standard drift, uneven expertise, and reliability concerns. Both approaches struggle to balance reliability with developmental value: instructor marking prioritizes consistency but scales poorly, whereas peer review promotes learning but sacrifices standard stability.

Consequently, factors such as scorer fatigue, cognitive load, standard drift, and cost escalation interact to create systematic challenges that are difficult to overcome within purely human-centered systems. This structural tension between reliability and developmental value has catalyzed growing interest in AI-supported intervention as a potential mediating mechanism. Rather than replacing human judgment, AI is increasingly positioned as a means to reconcile consistency with formative engagement.

### 2.3 Advances in automated and AI-supported scoring

The emergence of AES (automated essay scoring) has substantially reduced the burden of manual grading in large-scale standardized marking such as the TOEFL and GRE (Chan et al., 2022; Ke and Ng, 2019). AES systems generate relatively consistent scores based on linguistic and structural features (Li et al., 2022). Although AES can produce reliable scores highly correlated with human ratings in vocabulary, grammar, and discourse structure, research consistently shows that such systems perform more effectively on surface-level linguistic features than on deeper dimensions of meaning, reasoning, and originality (Yang et al., 2022).

Recent advances in large language models (LLMs) have extended the role of AI in marking, enabling systems to provide both numerical scores and rubric-aligned feedback. Empirical evidence has demonstrated that rubric-based AES tools can achieve reliability and validity comparable to human raters in English as a Foreign Language (EFL) writing contexts (Pack et al., 2024). Similarly, comparative studies of ChatGPT-generated scores and expert evaluations reveal a high level of agreement in surface features, yet studies increasingly report difficulties in evaluating higher-order aspects of writing (Wetzler et al., 2024).

While prior research has documented the technical reliability and efficiency gains of AES and large language model-based feedback systems, emerging scholarship increasingly questions whether such systems meaningfully support higher-order evaluative reasoning (Cotton et al., 2024). Research comparing AI and human evaluators finds that while AI-generated feedback is consistent and efficient in identifying mechanical and organizational problems, it shows limitations in deeper evaluation of argument structure and personalized revision strategies (Jovic et al., 2025). Moreover, existing applications frequently prioritize output optimization and feedback efficiency over the cultivation of higher-order evaluative capacities (Kasneci et al., 2023). Even in newer human-AI scoring frameworks, the main focus is still on scalability, cost reduction, and consistency, while comparatively less attention has been given to how students interact with AI during evaluation activities and how such interaction may shape evaluative reasoning processes. Much less attention is given to how AI feedback shapes students’ evaluative monitoring, standards internalization, or the development of evaluative skills.

### 2.4 The emergence of student–AI collaboration

Existing AI-supported assessment approaches can broadly be grouped into three forms: traditional human scoring, automated scoring systems, and emerging human–AI collaborative frameworks. Human evaluation emphasizes expert judgment, providing strong expertise, but struggles with scalability and timeliness. Automated scoring improves efficiency and consistency but often shows limitations in evaluating complex reasoning. Collaborative models attempt to combine the strengths of both approaches, offering integration of human and AI capabilities, yet their underlying learning mechanisms remain insufficiently theorized, reflecting a theoretical gap. Collaborative grading aims to integrate the consistency and scalability of AI with the evaluative expertise of human raters, rather than replace human judgment, thereby enhancing scoring reliability while preserving human interpretive authority. For instance, studies on hybrid frameworks for short-answer scoring show that integrating automated scoring with human review can reduce overall costs while maintaining quality and reliability (Funayama et al., 2022). In such hybrid models, AI rapidly generates rubric-aligned scores and formative feedback, while teachers or students critically engage with these outputs. This approach restructures the grading workflow by allocating evaluative responsibilities between automated systems and human reviewers, while preserving teachers’ or teaching assistants’ capacity to provide critical revisions and pedagogical insights (Lu et al., 2025).

Other scholars have proposed AI-driven marking frameworks that provide consistent and scalable evaluation for open-ended tasks, while emphasizing the necessity of maintaining human oversight at the final stage to safeguard educational value and evaluative depth (Ilieva et al., 2025). Within such human–AI interactive contexts, students may be more willing to experiment and revise, as they can iteratively optimize their writing through cycles of immediate AI feedback without the anxiety of direct instructor evaluation (Mohammed and Khalid, 2025). In this sense, student-AI collaboration is also associated with reduced evaluation anxiety and increased opportunities for iterative revision. These interaction cycles position AI not merely as a scoring instrument but as an intermediary that mediates learners’ engagement with evaluative standards and feedback processes. This highlights the potential of AI-assisted scoring and interaction to help students promptly identify and address weaknesses.

Despite these promises, research on collaborative scoring remains in its infancy. Existing studies are often limited to small-scale or discipline-specific contexts and constrained by narrow task types or scoring rubrics, which restrict generalizability. For example, the CoGrader project demonstrated benefits in efficiency and consistency but also revealed that such tools are insufficient to fully replace human judgment in tasks requiring creativity and applied knowledge (Zixin et al., 2025). Collaborative models appear to balance reliability and formative engagement at the operational level. However, they are seldom grounded in integrated psychological theory, and provide limited explanation of how AI-mediated interaction supports the sustained development of evaluative judgment over time. This calls for a psychologically grounded calibration framework that moves beyond efficiency and reliability logics, and instead conceptualizes student-AI collaboration as an interactive process in which students iteratively compare, revise, and refine their evaluative judgments through structured engagement with AI-generated feedback.

## 3 Methodology

### 3.1 AI-supported collaborative marking framework

Conventional AI systems, due to the lack of an expert-level knowledge base, often struggle to accurately capture the academic characteristics of scholarly writing. As a result, their evaluation outputs frequently deviate significantly from expert benchmarks, a gap that cannot be overlooked. Specifically, due to the nature of AI scoring algorithms, limitations in training datasets, the evolving nature of model parameters, and the inherent randomness of algorithmic outputs, AI-generated scores often lack consistency (Bui and Barrot, 2025). Moreover, individual differences in students’ knowledge bases and cognitive frameworks result in variability in scoring criteria. Some students apply lenient standards, while others are excessively strict, producing outlier scores that fall outside a reasonable range.

**Figure 1:**
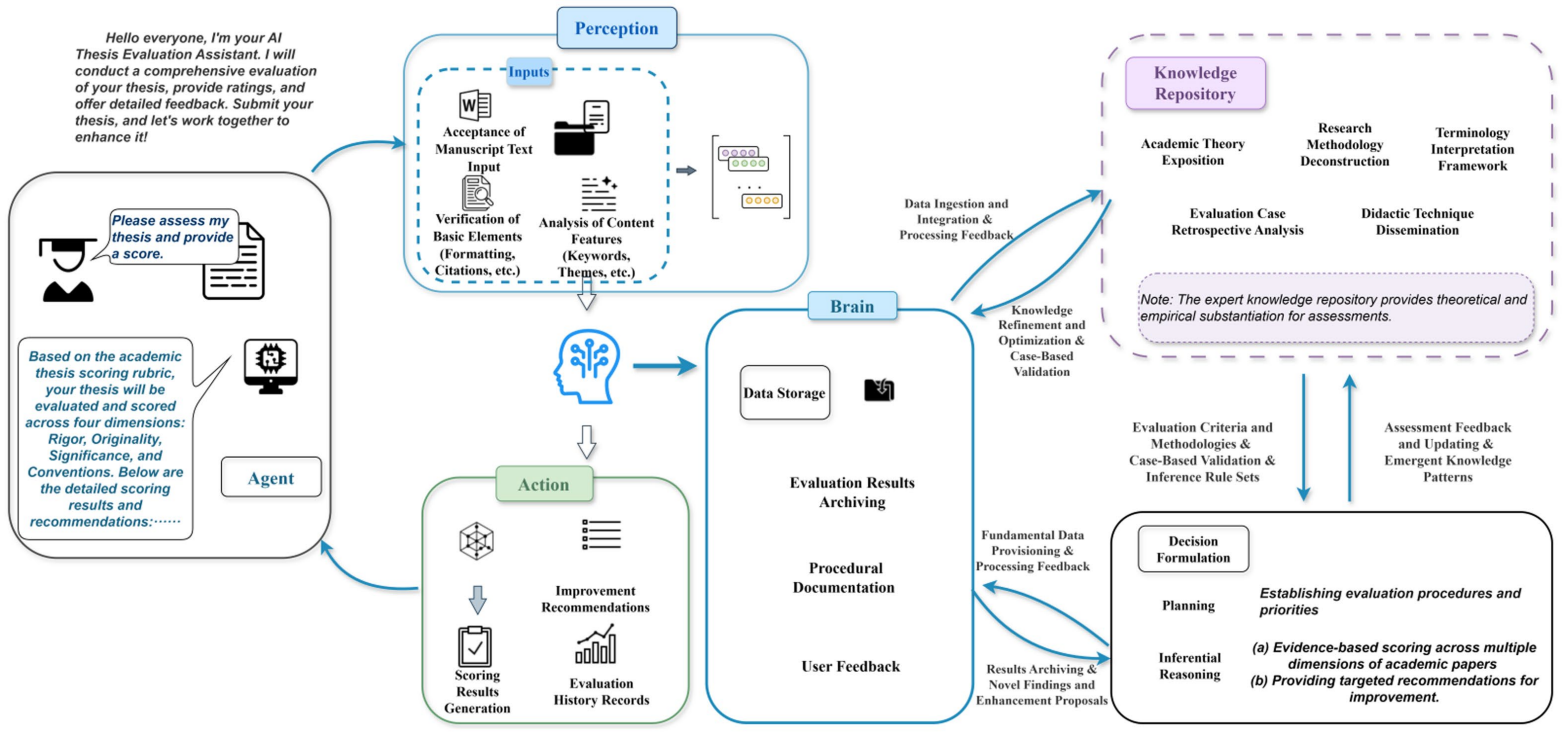
AI-supported collaborative marking framework.

To address these challenges, this study integrates an expert-informed knowledge base into an AI agent model and embeds it within a collaborative marking process, as illustrated in Figure 1. The collaborative framework positions students as active judges who interact with AI-generated feedback to monitor, reflect on, and recalibrate their evaluative decisions. Student participation thus serves a dual purpose: improving alignment with expert standards while simultaneously cultivating evaluative judgment, reflective reasoning, and self-regulated assessment skills.

The intelligent agent developed in this study for research paper assignment evaluation consists of three core modules identified as brain, perception, and action (Xi et al., 2025). The brain module manages information storage, knowledge organization, and decision formulation. Its storage unit records evaluation outcomes, operational processes, and user feedback, while the knowledge base integrates academic theory exposition, methodology deconstruction, terminology interpretation, case-based analysis, and didactic techniques. The decision formulation unit applies expert-embedded evaluation criteria, drawing on reasoning rules and models to generate evidence-based judgments on rigor, originality, significance, and conventions. The brain module continuously exchanges information with the knowledge base, which both constrains and updates decision processes. The perception module extends input beyond text to multimodal processing, analyzing manuscript content, formatting and citation compliance, thematic features, and visual elements such as figures and framework diagrams to assess structural coherence. The action module provides detailed evaluation scores, targeted recommendations, and visualized performance trajectories, supporting students in identifying areas for improvement and planning research. The expanding expert knowledge base enhances evaluation precision and feedback quality. In parallel, students critically examine the agent’s outputs, identify inconsistencies, and refine results, thereby improving the overall quality of evaluation and supporting graduate students’ academic development.

To address these reliability risks and reinforce ethical foundations, we implemented multiple safeguards: (a) automatic cross-checking of factual claims against an expert knowledge base, with discrepancies flagged for human review; (b) structured prompt templates requiring evidence statements, source attributions, and uncertainty indicators; (c) mandatory human adjudication for outputs exceeding uncertainty thresholds. These measures enhanced reliability and minimized unsupported inferences (Langer et al., 2024; de Teixeira Melo et al., 2025; Barandas et al., 2022). To further mitigate biases and ensure transparency, we de-identified all data, prioritized disciplinary and cultural balance in constructing the knowledge base, and employed a human-AI co-review mechanism that flags low-confidence or inconsistent outputs for verification. These measures enhance reliability, prevent undue influence from biased recommendations, and minimize unsupported inferences.

In this experiment, we used the DeepSeek-V3.1-Terminus version of deepseek-chat. Compared with other large language models, this version offers higher reasoning efficiency and stronger agentic capabilities, making it particularly suitable for academic text comprehension and evaluation. Its robust language understanding, reasoning, and explanatory capacity enable multidimensional marking of scholarly texts. Prompt engineering optimized task alignment. Prior research indicates that explicit task descriptions, role specifications, and constraints improve accuracy, consistency, and interpretability (Knoth et al., 2024; Korzynski et al., 2023; Marvin et al., 2024; Giray, 2023; Chen B. et al., 2025). Accordingly, prompts were designed to (a) specify evaluation dimensions and criteria, (b) require justifications for each score, and (c) ensure cross-paper comparability. The proposed AI Agent was developed on an ontology of scoring criteria driven by expert prior knowledge, integrating semantic representation of scoring dimensions with a cognitive model of exemplar texts. Through structured prompt engineering, the agent anchored DeepSeek’s reasoning to four core dimensions: rigor, originality, significance, and conventions. Constrained reasoning chains ensured adherence to rubric rules and contextual semantics. The resulting feedback demonstrated both symbolic interpretability, with comments explicitly linked to rubric items, and cognitive interpretability, allowing students to understand the causal connections between evaluative judgments and writing decisions. This mechanism transforms evaluation into a traceable, dialectical metacognitive scaffold, supporting evaluative literacy and critical self-monitoring skills.

In this study, the AI agent is conceptualized as a structured interaction support that may assist students in organizing evaluative tasks and reflecting on scoring decisions during the assessment process, and this conceptualization draws on metacognitive and cognitive load perspectives to interpret the potential role of AI-mediated feedback in shaping evaluation activities (Tsakeni et al., 2025; Yan et al., 2025; Fischer et al., 2024; Xu et al., 2025). Specifically, the system provides three categories of essential cognitive support: (a) monitoring cues, which facilitate error detection by highlighting discrepancies between students’ judgments and expert standards; (b) reflective reasoning prompts, which stimulate reflective processing by requiring learners to justify their scoring decisions and verify the coherence of their evidence chains; (c) cognitive load regulation, which reduces extraneous cognitive load by decomposing complex evaluative tasks into sequential and structured subcomponents, enabling learners to allocate more resources to conceptual integration and higher-order analysis.

With the aid of this AI-based scaffolding, students’ evaluative processes exhibit a more clearly articulated trajectory of psychological operations (Levin et al., 2025). To mitigate potential self-evaluation bias, students engaged in iterative cycles of self-assessment followed by comparison with AI-assisted and expert-informed feedback. This process helped students calibrate their judgments, identify discrepancies, and refine their evaluations systematically. Students first formulate an initial judgment and, upon receiving AI-generated feedback, initiate metacognitive monitoring to identify divergences between their own judgments and expert benchmarks (Händel et al., 2020). During the subsequent recalibration phase, learners adjust their use of evidence, reasoning pathways, and scoring decisions, adopting alternative strategies when necessary. Through repeated feedback cycles, these processes are continually reinforced. Over time, students acquire a more functional understanding of how evaluative criteria operate, achieving a shift from external scaffolding to internal representations (Ibarra-Sáiz et al., 2025). The internalization of criteria may reflect a gradual increase in students’ familiarity with how evaluation criteria are interpreted and applied in academic assessment contexts. Repeated comparison with AI-generated feedback may help students become more familiar with evaluation criteria and apply them more consistently during scoring activities.

Through this iterative interaction pattern, the AI system supports students in engaging in higher-level cycles of monitoring, reflection, and adjustment, thereby strengthening their self-regulatory capacity in complex academic tasks and enhancing both the quality and stability of their evaluative judgments.

### 3.2 Data collection and scoring criteria

This section describes the dataset used in the study, including its composition, participant characteristics, and the criteria applied for evaluation. A comprehensive understanding of the current state of students’ academic evaluation practices, particularly the challenges faced by graduate students, serves as the foundation for developing and applying AI-based models. To this end, research paper assignments were collected each academic year from the Artificial Intelligence in Education course offered by the School of Teacher Education at Hubei University between June 2020 and June 2025 through an online intelligent education platform. At the end of each academic year, the course instructor required students to conduct self-evaluations of their own research papers. The resulting dataset included 121 research paper assignments written by first-year master’s students enrolled in the course, along with their corresponding self-evaluation records. A total of 121 graduate students participated as student evaluators, including 28 males (23.14%) and 93 females (76.86%). All data were rigorously collected during the in-class writing and evaluation process, and all participants possessed a basic level of academic writing competence. As part of the course requirements, each student evaluator was instructed to complete an approximately 8,000-word short research paper following standard academic writing guidelines. The assignment required students to demonstrate a clearly defined research question, appropriate research methodology, and coherent academic reasoning. Students were provided with access to exemplar papers and clear evaluation criteria aligned with expert benchmarks, allowing them to compare their judgments and adjust scoring where necessary. All papers were later used in the experimental procedures described in Section 3.3. Prior to the study, all participants were fully informed of the research purpose and their right to withdraw at any time. They were assured that all data would remain confidential and be used solely for research purposes. The study protocol was reviewed and monitored by the Institutional Review Board (IRB) of Hubei University.

Although definitions of research quality in academic writing may vary, methodological plausibility or reliability, originality or novelty, and scientific or societal value are commonly included or explicitly defined as three core attributes (Langfeldt et al., 2020). Similarly, the UK Research Excellence Framework (REF) defines research quality through the dimensions of rigor, originality, and significance. In this study, we adopted the REF’s national evaluation criteria^1^ as a standard used by both human raters and generative artificial intelligence to assess students’ writing. Building on prior scholarship, we made targeted adaptations to the original REF criteria to better align with the specific requirements of the academic writing evaluation task (Tomaszewski et al., 2020; Alajami, 2020). Given the prevalent lack of adherence to academic writing conventions among graduate students, there is an urgent need to establish explicit marking criteria for writing conformity. Such criteria can offer clear guidance and evaluative standards to help students align their work with accepted academic norms. By emphasizing accuracy in formatting, citation practices, and language use, conformity-based marking encourages students to develop greater attention to detail and fosters improvements in the overall quality of academic writing (Rivera, 2022). The guidance for students is ultimately structured around four dimensions: rigor, originality, significance, and conventions. Table 1 presents the mapping between the rubric dimensions and the corresponding feature variables. Each dimension was equally weighted at 25%. The overall paper quality score was calculated as the arithmetic mean of the four dimension scores, yielding a total score ranging from 0 to 100.

**Table 1:** Mapping between research paper assignment scoring rubric dimensions and corresponding characteristic variables.

**Primary Indicators**	**Score**	**Secondary Indicators**	**Description of Indicators**
Rigor	100	Research design	The research design is methodically rigorous, featuring a systematized data collection process, with potential biases anticipated and controlled through appropriate measures.
		Data analysis	Statistical methods are appropriate and reproducible, with full transparency via open disclosure of data and code to ensure replicability and rigor.
		Argumentation logic	Conclusions are grounded in ample empirical evidence, forming a coherent and tightly reasoned logical chain that upholds methodological integrity.
Originality	100	Originality in topic selection	The study is situated within an emerging and under-explored domain with the potential to generate significant insights and open new avenues for scholarly investigation.
		Originality in problem identification	The research identifies gaps or limitations within existing paradigms and proposes a novel direction that challenges conventional lines of inquiry.
		Methodological innovation in addressing the research problem	The study introduces original tools, frameworks, or methodological approaches, or applies existing methods in a novel context to yield fresh perspectives.
		Originality of research findings	The findings advance the field by offering new theoretical or empirical contributions that question or reconceptualize established paradigms.
Significance	100	Scholarly value	The study contributes to the advancement of social science theories or fosters interdisciplinary integration and application across social science domains.
		Practical value	The research offers evidence-based insights to inform policy-making or demonstrates practical relevance in addressing real-world societal challenges.
		Societal value	The findings support the development of social welfare systems and the enhancement of public service delivery, with potential impact on community wellbeing.
Conventions	100	Citation standards	References are cited consistently throughout the text; the reference list is properly ordered and formatted according to prescribed guidelines; sources cited are of high scholarly quality and relevance.
		Formatting requirements	Content is logically organized into appropriate sections; headings and body text follow a consistent style across hierarchical levels; punctuation and numeral usage comply with language-specific conventions in both Chinese and English.
		Textual precision and presentation	Language use (including Chinese and English) is accurate and appropriate; word or character counts meet the minimum required; tables and figures are presented, properly labeled, and placed in the correct locations within the text.

### 3.3 Experimental procedure

This section outlines the experimental procedures applied to the dataset described above, detailing the evaluation conditions, rater assignments, and overall study design focusing on student–AI interaction and cognitive processes. This study employed a mixed-methods design, integrating a quantitative analysis with a qualitative analysis. The quantitative component involved within-subject comparisons across four evaluation conditions: student-only, AI-only, AI with RAG, and AI-supported collaborative marking. The qualitative component emphasized coding and sequential analysis of students’ interactions with the AI agent. This design is consistent with the mixed-methods framework (Creswell and Plano Clark, 2011).

The experimental procedure was organized into two stages to examine both evaluation outcomes and interaction processes. In the first stage, the full dataset of 121 research papers was used to conduct quantitative comparisons across four evaluation conditions: student-only evaluation (*N*_s_ = 121), AI without RAG (*N*_a_ = 1), AI with RAG (*N*_r_ = 1), and expert benchmarking. Each of the 121 students conducted a self-evaluation of their own paper using the same standardized rubric, while the same set of 121 papers was also evaluated by a general AI model, a RAG-enhanced AI model, and an expert rater. This design enabled direct comparison of scoring consistency and deviations across human and AI-based evaluation conditions. During the subsequent evaluation activity, each student evaluator was required to: (1) assess their own paper using the provided scoring rubric across four dimensions—rigor, originality, significance, and convention; (2) assign a numerical score (0–100) for each dimension and calculate a total score based on the average of the four dimensions; and (3) provide a brief explanation (at least 40 words) for each dimension score. To reduce potential self-evaluation bias, the evaluation process was structured using clearly defined scoring rubrics and AI-supported collaborative marking interactions. Students were encouraged to justify their scoring decisions and compare them with AI-generated feedback based on the expert knowledge base. This structured interaction was designed to promote reflective judgement. In addition to student self-evaluations, two AI-based marking systems were employed. The first was a general AI model that generated evaluation results directly from the paper and rubric. The second was a retrieval-augmented generation model (AI with RAG), which incorporated an expert-informed knowledge base containing scoring criteria and exemplar papers to enhance evaluation accuracy. In both AI-only conditions, students did not interact directly with the AI systems; instead, the generated outputs served as comparative baselines. In the AI-supported collaborative marking condition, students and the AI jointly participated in the evaluation process, with the AI drawing on the same expert-informed knowledge base.

**Figure 2:**
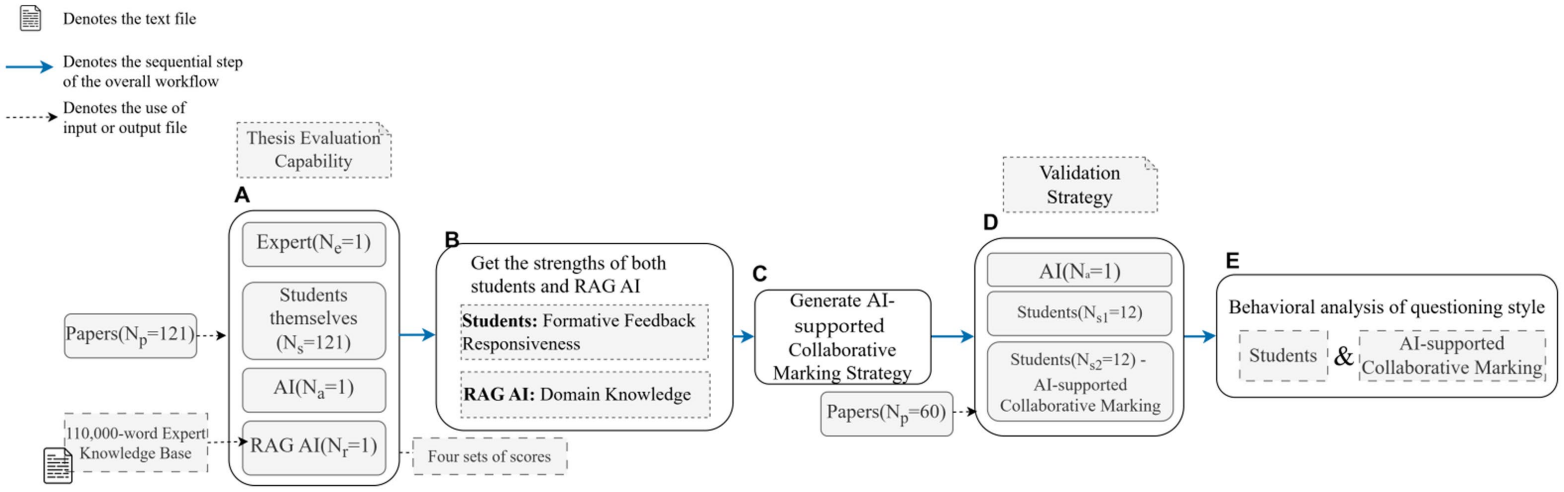
Experimental procedure.

In the second stage, interaction processes during evaluation were examined through a smaller structured task. From the existing pool of 121 students, 24 students were randomly selected. Twelve students evaluated a subset of 60 papers drawn from the original dataset of 121 papers independently using the rubric, while the other 12 completed the same evaluations with assistance from the AI agent. Each student evaluated five papers, ensuring comparable workloads across groups. Interaction logs generated in the AI-assisted condition were recorded and later used for behavioral coding and Lag Sequential Analysis. For the AI-based conditions, one general AI model and one RAG-enhanced AI model each served as a single evaluator (*N* = 1 for each AI system). The detailed experimental procedure is illustrated in Figure 2. Figure 2 presents the experimental workflow and the sample sizes (*N* values) involved at each stage of the study to facilitate clearer understanding of the procedure.

Furthermore, an expert evaluator with over 10 years of experience in academic writing instruction provided benchmark scores for all 121 papers at the end of each course cycle. This rater held a doctoral degree and had nearly 10 years of experience in evaluating research paper assignment at both undergraduate and graduate levels. Prior to the formal evaluation, the expert reviewed the standardized scoring rubric and several exemplar papers to calibrate the interpretation of the evaluation criteria. To examine scoring stability, a subset of papers was re-evaluated by the same expert after a two-week interval. The consistency between the two scoring rounds was examined using the intraclass correlation coefficient (ICC), which indicated high intra-rater reliability (ICC = 0.937). The involvement of this expert rater substantially reduced variability in the scoring process and ensured consistency of standards across the four comparative conditions: AI without RAG, student-only evaluation, AI-supported collaborative marking, and expert evaluation. Since multiple raters often introduce interpretive differences in scoring standards, which may lead to inconsistencies in human evaluation, this study minimized such risks by employing a single senior rater. This approach maintained a uniform benchmark and facilitated meaningful comparison across different evaluation scenarios (Bui and Barrot, 2025).

In this study, the AI agent provided structured feedback designed to support students’ monitoring of their own judgments, reflective reasoning, and internalization of evaluative criteria. The AI agent we designed evaluated each paper by integrating marking across 13 key workflow components, which collectively captured multiple dimensions of paper quality. Specifically, the study conducted a quantitative analysis of 121 graduate papers, focusing on four primary dimensions and the overall mark. In total, 7,865 evaluation records were collected, and all records were included without omission to ensure the completeness and robustness of the findings.

Ultimately, the collaborative marking process emphasized student–AI interaction as a means to strengthen evaluative judgment, reflective reasoning, and self-regulated learning. Upon completion of the evaluation, all student and AI assessment data were integrated into a standardized database to facilitate analysis of cognitive processes and performance outcomes. The study adhered strictly to ethical guidelines, including obtaining informed consent from all markers, ensuring confidentiality and anonymity of information, and safeguarding participants from potential harm.

### 3.4 Behavioral coding and lag sequential analysis

To closely examine students’ behavioral characteristics during AI-supported academic evaluation, we collected dialogue logs between students and an expert-knowledge–based AI agent after the paper-assessing tasks were completed. This enabled the capture of temporal interaction patterns reflecting students’ cognitive and metacognitive engagement during evaluation. Participants were recruited through convenience sampling at a comprehensive university.

This methodological approach was grounded in cognitive and educational psychology, as dialogic interaction data allow fine-grained observation of metacognitive monitoring, evaluative judgment formation, and regulatory decision-making processes unfolding over time. To characterize students’ interaction behaviors, we employed the collaborative problem-solving model to design a coding framework and conducted qualitative content analysis (Hesse et al., 2014; Wu et al., 2024).

Two trained graduate students participated in the behavioral sequence annotation. Prior to formal coding, both coders completed a structured training protocol, which included: (a) reviewing the theoretical constructs underlying the coding scheme—evaluative judgment, metacognitive monitoring, reflective reasoning, and cognitive regulation; (b) jointly coding a pilot dataset comprising 15% of the interaction logs; and (c) refining the codebook through iterative discussion. Inter-rater reliability during the pilot phase reached 0.916. The intercoder reliability was calculated using Cohen’s Kappa, which is widely recommended for categorical behavioral coding (Mchugh, 2012; Viera and Garrett, 2005). A Kappa value of 0.80 or above is generally considered indicative of strong agreement, and our coefficients (0.916 in the pilot phase, 0.892 in the final coding) meet this threshold, confirming the reliability of the behavioral annotations (Koch, 1977). Discrepancies were resolved through discussion, and unresolved cases were adjudicated by a senior researcher.

After reliability was established, the two coders independently coded the interaction behaviors of 12 students during AI-supported collaborative marking of 60 papers. The intercoder reliability coefficient reached 0.892, indicating strong agreement. In total, 269 events were coded, of which 101 were generated by junior graduate students and 168 by senior graduate students.

Table 2 presents the coding scheme, developed based on prior work (Heikkinen et al., 2024; Romeo and Conti, 2025; Pavlović, 2025; Wahn et al., 2023; Cheng et al., 2025; Shields et al., 2024; Vo et al., 2024; Onan et al., 2024), to characterize behavioral processes in expert-knowledge–based AI-supported academic evaluation. The behavioral categories were theoretically grounded in self-regulated learning and evaluative judgment frameworks, conceptualizing academic marking as a cognitively demanding problem-solving activity involving forethought, monitoring, and reflective regulation. Specifically, behaviors in the Problem Analysis phase capture goal setting, task interpretation, and distributed metacognition; Information Seeking behaviors reflect monitoring of knowledge gaps and strategic control under uncertainty; and Monitoring and Evaluation behaviors index reflective reasoning, criteria calibration, and cognitive regulation. This mapping allows interaction traces to be interpreted as psychologically meaningful indicators of students’ evaluative judgment development within human–AI collaboration contexts.

**Table 2:** Coding scheme for AI-supported collaborative marking interaction behaviors.

**Phase**	**Category**	**Code**	**Description**	**Example**	**Self-regulated learning mechanisms**
Problem analysis	Student command	SC	The student initiates an evaluation task; the AI Agent immediately responds with a preliminary assessment and scores.	Please evaluate this paper in terms of Rigor, Originality, Significance, and Conventions, and justify.	Planning and goal setting
	Repetition	RE	The student repeats the previous command without any modification.	/	Automation bias
	Defining the role	DC	The student assigns the machine a specific role as an academic assistant and clarifies the evaluation needs.	As my academic assistant, please help me improve the evaluation of this paper.	Distributed metacognition
	Sharing information	SI	The student provides the agent with background materials relevant to the evaluation task.	Uploading relevant academic literature or research data.	Cognitive offloading
Information seeking	Asking a basic question	AR	The student asks the agent for basic knowledge or clarification about a specific topic.	Do you know how generative AI is applied in educational technology?	Information seeking
	Inquiring for ideas	II	The student requests suggestions or a possible evaluation approach from the agent without a clear initial idea.	Do you have any suggestions for structuring or conducting an evaluation for this paper on generative AI in educational technology?	Metacognitive control
Monitoring and evaluation	Identifying problems	IP	The student points out deficiencies or flaws in the generated content and suggests how to revise them.	The evaluation is too general; it needs to analyze specific examples from the paper more thoroughly.	Refutation and reflective reasoning
	Offering improvements	OI	The student proposes refinements or enhancements to the existing evaluation content.	Could you add more detailed descriptions under the conventions section of the evaluation?	Cognitive regulation and elaboration
	Requesting Explanations	EX	The student asks the agent to elaborate or clarify the rationale behind a particular score or evaluation.	Please provide a detailed explanation for the significance score and expand on the justification.	Metacognition and conceptual regulation

After completing coding, we conducted Lag Sequential Analysis (LSA) using GSEQ 5.1 to identify above-chance interaction patterns between students and the AI agent (Bakeman and Quera, 2011). Ordered behavior pairs were constructed to generate transition frequency matrices, from which transition probabilities were derived. Adjusted residuals (*z*-scores) were calculated to determine whether observed transitions exceeded expected values. Only transitions with |*z*| ≥ 1.96 were considered statistically significant, and significant patterns were visualized as behavioral transition diagrams for each student cluster (O’Connor, 2000).

## 4 Results

This section systematically presents the application outcomes and key findings of AI-supported collaborative marking strategies in academic mentoring. All statistical analyses reported in this section were conducted using SPSS. Descriptive statistics were first calculated to quantify discrepancies between expert evaluations and alternative rating sources, including mean absolute error (MAE), root mean square error (RMSE), and mean absolute percentage error (MAPE). In addition, Pearson correlation analyses were performed to examine the associations between expert ratings and ratings generated under different evaluation conditions. Paired-sample *t*-tests were further conducted to assess systematic differences between expert benchmarks and alternative rating approaches, with 95% confidence intervals reported to indicate the precision of the estimated differences. The normality assumption of the paired differences for each dimension was examined using Shapiro–Wilk tests, and no significant deviations from normality were observed, supporting the use of paired-sample *t*-tests. Effect sizes for the paired comparisons were estimated using Cohen’s d to indicate the magnitude of score differences. First, by comparing student-led evaluations with those conducted through RAG-based Agent collaboration, we confirmed the significant advantages of collaboration in enhancing the accuracy and consistency of marking, highlighting its distinctive value across multiple dimensions, including rigor, originality, significance, and conventions. Second, focusing on changes in students’ inquiry behaviors under AI-supported guidance, we analyzed the behavioral characteristics and transitions observed among students from diverse academic backgrounds, demonstrating the Agent’s potential role in shaping academic reasoning and evaluative competence. Finally, a detailed comparison among AI without RAG, AI-supported collaborative marking, and expert evaluations further underscored the effectiveness of collaborative strategies in simulating expert scoring patterns and optimizing marking outcomes, offering empirical support and preliminary actionable insights for academic mentoring practices.

### 4.1 Improvement in accuracy and consistency of academic evaluation through AI-supported collaborative marking

**Figure 3:**
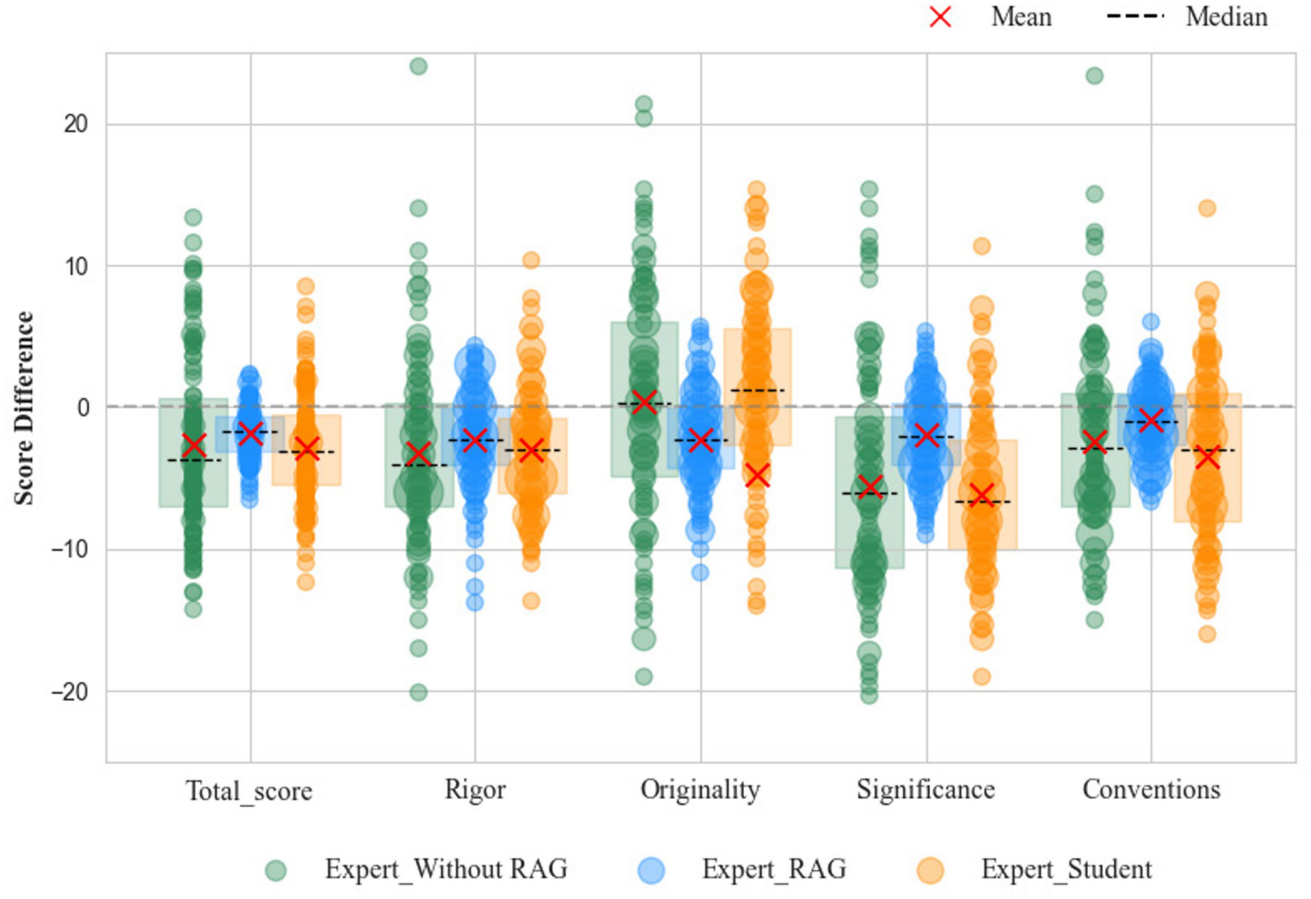
Evaluation results of students, AI without RAG, AI with RAG, and experts on overall score, rigor, originality, significance, and conventions.

To address RQ1, the results demonstrate that AI-supported collaborative marking substantially improved the accuracy and consistency of academic evaluation compared with student-only marking, with outcomes aligning more closely to expert benchmarks. As shown in Figure 3, within an authentic academic marking dataset, the median student scores on the rigor dimension were markedly below zero, with a considerable number of negative outliers. This indicates that students tended to assign lower ratings than experts with respect to the logical structure, argumentative coherence, and methodological rigor of scholarly work, revealing pronounced and consistent discrepancies. This pattern suggests insufficient calibration between students’ internal evaluative standards and expert criteria, reflecting limitations in monitoring judgment accuracy during complex analytical evaluation. For the originality dimension, the distribution of score differences was more dispersed, with a median slightly above zero, suggesting substantial individual variability among students in recognizing novel contributions and a lack of uniform evaluative standards at the cohort level. This dispersion implies that students relied on heterogeneous and often implicit heuristics when judging originality, indicating underdeveloped evaluative judgment and inconsistent application of criteria. In terms of significance, student ratings were overall lower than expert ratings, with a median below zero and a broad dispersion, implying a limited perspective in appraising the scholarly value and societal impact of research outputs. This finding reflects difficulties in integrating disciplinary relevance and broader impact into evaluative reasoning, a process that requires higher-order abstraction and reflective judgment. For the conventions dimension, the score differences showed a median below zero with bidirectional outliers, reflecting uneven competencies among students in evaluating fundamental aspects such as formatting, academic language, and citation practices, and indicating overall low consistency. This pattern suggests fragmented procedural knowledge and weak self-monitoring when applying explicit academic norms.

Table 3 presents quantitative indicators of the deviations between student and expert ratings. The calculated MAE was 4.152, the RMSE was 4.901, and the MAPE was 0.053. Although these values suggest that the average deviation falls within an acceptable range, the variability of the errors highlights instability in student evaluative standards, with some students showing substantial divergence from expert judgments. Psychologically, such instability indicates that students’ evaluative reasoning has not yet been fully internalized and remains vulnerable to contextual fluctuations and judgment bias. Collectively, these findings suggest that students face challenges in achieving expert-level marking accuracy across the four dimensions of rigor, originality, significance, and conventions. While moderate alignment with expert ratings was observed in rigor and originality, student scores tended to fluctuate toward either inflation or deflation, and notable gaps were particularly evident in significance and conventions, underscoring the uneven development of evaluative judgment across different cognitive demands.

**Table 3:** Differences in overall evaluation scores among students (*N* = 121), AI without RAG (*N* = 1), AI with RAG (*N* = 1), and expert (*N* = 1).

**Indicator**	**Students**	**Without RAG**	**RAG**
MAE	4.152	5.741	2.140
RMSE	4.901	6.666	2.582
MAPE	0.053	0.074	0.028

To address these cognitive challenges, the expert-informed AI system was designed to make evaluative criteria explicit and comparable, thereby supporting students’ monitoring and recalibration processes. The repository systematically consolidates conceptual expositions, methodological analyses, and technical terminology, along with insights distilled from previous marking cases and practical techniques. The resulting database, comprising approximately 110,000 words, is deeply integrated with a generative artificial intelligence system. The system functions as a reference framework that externalizes expert standards for student comparison. The resulting model demonstrated strong performance across critical evaluation tasks, including originality detection, significance appraisal, and compliance auditing. It effectively supports students in narrowing performance gaps with expert evaluators by facilitating reflective comparison and evidence-based adjustment of judgments, contributing to the development of evaluative literacy.

Quantitative analysis revealed that when students were assessed against outputs generated by conventional computational models without access to the expert repository, significant discrepancies emerged across all rating dimensions, reflecting low consistency with expert judgments. By contrast, the agent enhanced with RAG exhibited markedly superior alignment, producing median scores closer to expert benchmarks and displaying tighter score distributions. The reduced dispersion indicates improved standard calibration and more systematic evaluative reasoning. Particularly in domains of originality, significance, and adherence to conventions, the enhanced agent’s ratings were more concentrated, showed reduced outliers, and demonstrated minimal deviation from expert standards. For example, the AI-without-repository group yielded the highest mean absolute error (MAE = 5.741), while the RAG-enhanced agent achieved a substantially lower error (MAE = 2.140). Similar patterns were observed in RMSE and MAPE. Findings also indicated that models lacking RAG support were more prone to repetitive or fabricated outputs.

To validate the efficacy of AI-supported collaborative marking, an observational study was conducted. Participants included junior graduate students, defined as first-year master’s students with limited prior experience in academic evaluation, and senior graduate students, defined as second-year or above master’s students with previous exposure to research assignments and academic marking tasks. Both groups had similar GPA and academic achievement levels, and due to the emerging nature of AI at the time, their prior exposure to AI tools and applications was minimal and comparable. They were tasked with reading and evaluating 60 papers drawn from a real-world academic marking dataset, both independently and in collaboration with the enhanced agent. Each paper was reviewed twice—once scored solely by the student and once through a student-Agent interaction. Even in the student-only condition, marking rubrics were provided to reflect authentic evaluative practice.

According to prior research (Brimi, 2011; Starch and Elliott, 1912), human-assigned scores on a 100-point scale typically exhibit an error margin of 35–40 points, prompting scholars to recommend coarse-grained scoring frameworks to mitigate such variability. More recent studies suggest that a deviation within 5 points from expert ratings may indicate strong agreement (Dubois and Lhotte, 2023). Drawing on Cicchetti and Koo’s interpretations of the intraclass correlation coefficient (ICC) (Cicchetti, 1994; Koo and Li, 2016), this error range typically corresponds to ICC values between 0.60 and 0.75, indicating reliable scoring. Therefore, in this study, scoring accuracy is evaluated based on a benchmark of no more than 5 points’ deviation from expert ratings.

**Figure 4:**
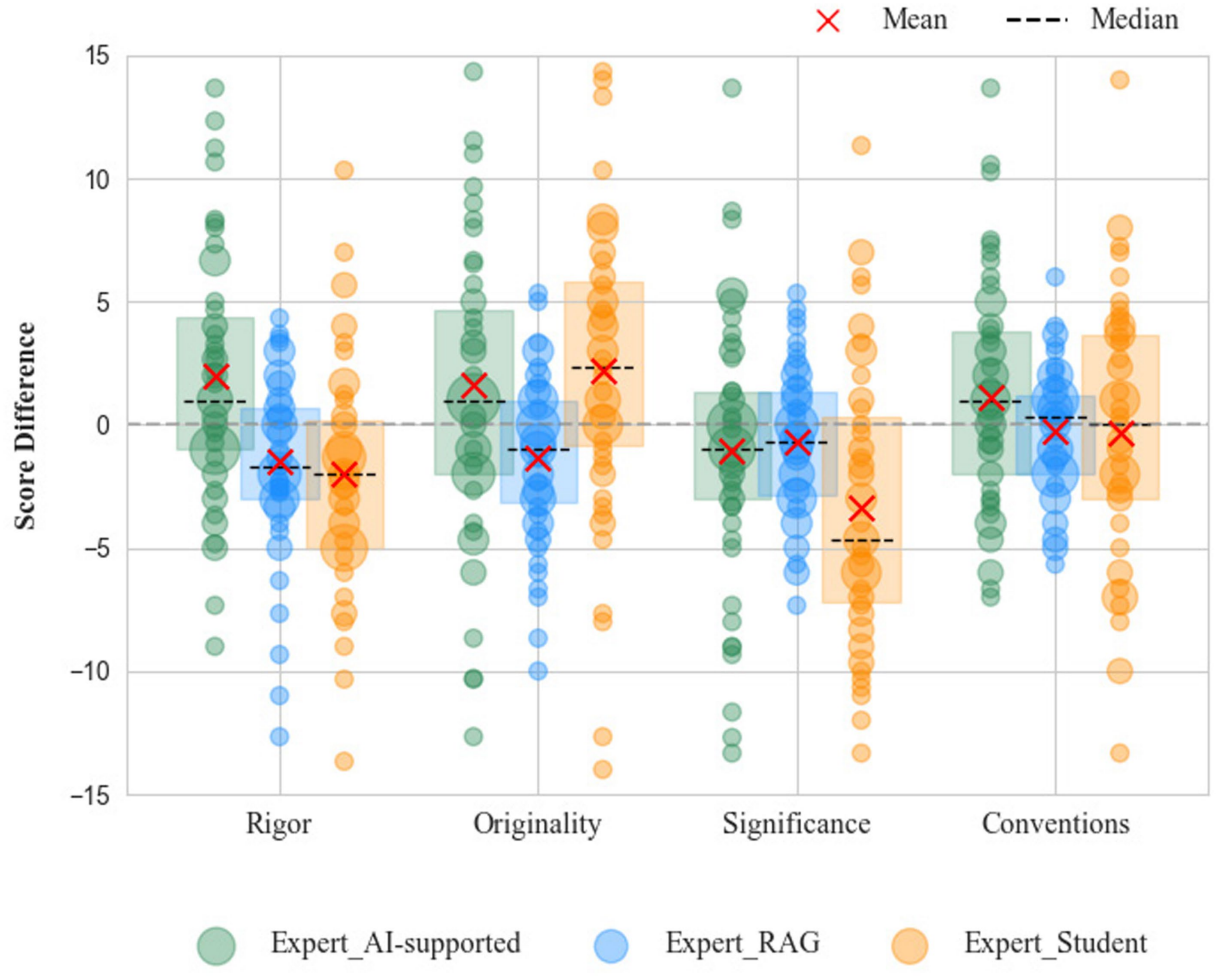
Marking results of students, AI-supported collaborative marking, and experts across overall score, rigor, originality, significance, and conventions.

Table 4 presents the mean absolute error (MAE = 3.260) for the student-only group, indicating a noticeable divergence from expert ratings. In contrast, the Student-AI Agent collaborative group achieved ratings more closely aligned with experts (MAE = 1.221), with similarly reduced RMSE and MAPE, demonstrating substantial gains in accuracy. Figure 4 further illustrates the score distributions across four key dimensions—rigor, originality, significance, and adherence to conventions. Compared to student-only ratings, the collaborative group’s markings clustered more closely around expert judgments, with tighter dispersion. This convergence reflects strengthened metacognitive monitoring and reflective judgment, as students adjusted their evaluations in response to explicit feedback and benchmark comparison. Specifically, scores for rigor and conventions were relatively higher, originality ratings were more convergent with expert evaluations, and significance ratings, though showing occasional deviations, remained more consistent overall. These findings highlight that the collaborative marking process, integrating retrieval-augmented data analysis with students’ evaluative judgment, effectively mitigates rating bias and strengthens scoring reliability. Importantly, the observed improvements suggest not only enhanced performance outcomes but also the gradual development of students’ evaluative reasoning and self-regulatory capacity.

**Table 4:** Differences in overall marking scores among students (*N* = 24), AI-supported collaborative marking (*N* = 24), and expert (*N* = 1).

**Indicator**	**Students**	**AI-supported collaborative marking**
MAE	3.260	1.221
RMSE	3.893	1.445
MAPE	0.042	0.016

The advantages of the collaborative model can be attributed largely to the retrieval-based feedback mechanism, which reinforces alignment with expert benchmarks. By comparing student ratings against a curated expert knowledge base, the system corrects scoring deviations in real time and delivers targeted suggestions for refinement. Students, in turn, adjust and optimize their evaluation strategies through iterative feedback, a process that reflects enhanced metacognitive monitoring, criterion calibration, and reflective evaluative reasoning rather than mere score correction. Concretely, the system provides: (1) in-depth analysis of logical structure, argumentation, and methodological rigor; (2) comparative marking of originality through cross-referencing with existing literature to identify novel contributions and potential overlaps; (3) contextualized evaluation of significance informed by disciplinary trends and broader academic impact; and (4) systematic checks on format, citation accuracy, and presentation standards. Over time, this collaborative approach not only enhances the quality of single-task marking but also supports students’ sustained development of evaluative expertise, facilitating the gradual internalization of expert standards and the transition from externally guided judgment to more self-regulated evaluation, fostering more structured and evidence-based marking practices.

Nonetheless, three primary challenges were identified: strengthening students’ capacity for critical analysis, improving concordance with expert ratings, and reducing evaluative workload to increase efficiency and pedagogical value. Compared with student-only or non-retrieval-based approaches, the collaborative model demonstrated stronger performance across dimensions, though occasional outliers were observed, particularly in rigor. Overall, the model showed potential to support the development of students’ evaluative literacy by stabilizing judgment criteria, reducing cognitive bias in complex evaluation tasks, and supporting sustained self-regulatory engagement through systematic feedback.

### 4.2 AI-supported collaborative marking outperforms AI without RAG and demonstrates closer alignment with expert evaluations

Regarding RQ2, quantitative analysis indicate that evaluation outcomes from AI-supported collaborative marking showed higher agreement with expert ratings than those produced by AI without RAG model, narrowing the performance gap in rigor, originality, significance, and conventions, suggesting that AI-supported collaboration supports more stable evaluative reasoning and criterion-consistent judgment. Based on the application of AI-supported collaborative marking strategies, we compared the characteristics and performance of AI without RAG and AI-supported collaborative marking against expert evaluations in the context of academic marking.

To examine the degree of alignment between expert ratings and those generated by AI without RAG across multiple dimensions, Pearson correlation analyses were conducted for overall scores and four key evaluation dimensions: rigor, originality, significance, and conventions. Subsequently, paired-sample *t*-tests were performed to further investigate differences. Tables 5, 6 present the correlations between expert and AI without RAG scores, which were generally low across all dimensions. The paired *t*-test results revealed that only in the dimension of originality was the difference between AI and expert ratings statistically non-significant (*p* = 0.004), whereas differences in overall score, rigor, significance, and conventions were all statistically significant. The analysis revealed a negative correlation between AI-generated scores and expert evaluations. This finding is consistent with certain prior studies reporting very weak, negative, or near-zero correlations between AI and expert marking (Verma et al., 2025). This result does not necessarily indicate a contradictory relationship but rather reflects the instability of the non-RAG model adopted in this study. In the absence of retrieval-augmented grounding or access to expert-derived reference materials, the AI system primarily relied on probabilistic text generation when evaluating academic quality. Consequently, its scoring occasionally deviated from expert standards, resulting in opposite trends between AI and expert evaluations, which implies that such models offer limited support for students’ evaluative calibration and may reinforce unstable or superficial judgment strategies when used as standalone references. These results suggest that although AI systems can produce seemingly coherent comments, their lack of factual grounding and contextual understanding may undermine consistency when judging complex academic writing tasks.

**Table 5:** Correlation analysis between expert ratings and student ratings without RAG across all dimensions.

Expert ratings	**Without RAG_Total**	**Without RAG_Rigor**	**Without RAG_Originality**	**Without RAG_Significance**	**Without RAG_Conventions**
E_Total	−0.104	−0.219	0.039	−0.135	−0.049
E_Rigor	0.06	−0.052	0.11	0.071	0.054
E_Originality	−0.093	−0.15	0.071	−0.22	0.011
E_Significance	−0.063	−0.169	0.05	−0.054	−0.07
E_Conventions	−0.318*	−0.410**	−0.193	−0.247	−0.249

*p < 0.05; **p < 0.01; ***p < 0.001.

**Table 6:** Summary statistics of score agreement between experts and ratings without RAG.

Measure	**Pearson *r***	**Mean**	**SD**	***t*-value**	***p***	**95% Confidence Interval** **(CI) for Indirect Effect**	**Cohen’s *d***
Expert-without RAG total	−0.104	−0.087	6.391	−0.105	0.917	[−1.738,1.564]	−0.014
Expert-without RAG rigor	−0.052	−0.888	6.939	−0.992	0.325	[−2.681,0.904]	−0.128
Expert-without RAG originality	0.071	3.150	8.251	2.957	0.004**	[1.019,5.281]	0.382
Expert-without RAG significance	−0.054	−2.327	8.193	−2.200	0.032*	[−4.444,-0.211]	−0.284
Expert-without RAG conventions	−0.249	−0.591	6.699	−0.683	0.497	[−2.321,1.140]	−0.088

*p < 0.05; **p < 0.01; ***p < 0.001.

**Figure 5:**
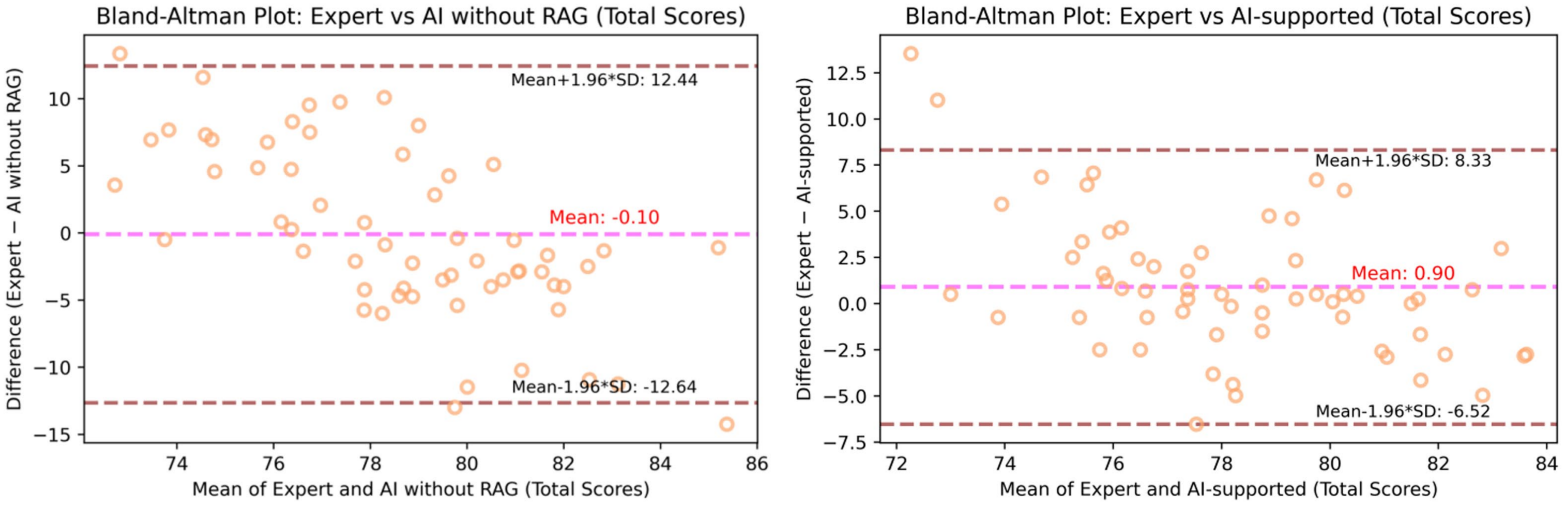
**(A)** Bland–Altman plot of overall score agreement between experts and AI ratings without RAG. **(B)** Bland–Altman plot of overall score agreement between experts and AI-supported collaborative marking ratings.

Furthermore, the visualization in Figure 5A illustrates that while most score discrepancies fall within an acceptable range, some scores exhibit substantial deviations, particularly at the overall score level. The 95% confidence interval of the mean difference is relatively wide, indicating limited consistency in scoring. These findings suggest that the AI without the RAG evaluation system has not yet reached expert-level performance and remains insufficient in reliably assessing student writing. In particular, originality and conventions require targeted optimization to better align with expert criteria.

To evaluate the degree of correspondence between expert markings and those obtained through AI-supported collaborative marking, we again conducted Pearson correlation analyses and paired-sample *t*-tests across the same five dimensions. Tables 7, 8 present the correlations between expert scores and AI-supported collaborative marking scores, which were consistently higher across dimensions. The t-test results further showed that for overall score, rigor, and significance, the ratings generated through collaboration were significantly consistent with expert judgments, while the differences in originality and conventions approached significance, indicating improved criterion alignment and more coherent evaluative reasoning under collaborative conditions.

**Table 7:** Correlation analysis between expert ratings and AI-supported collaborative marking ratings across all dimensions.

Expert ratings	**AI-supported Collaboration_total**	**AI-supported collaboration_rigor**	**AI-supported collaboration_originality**	**AI-supported collaboration_significance**	**AI-supported collaboration_conventions**
E_Total	0.415**	0.238	0.346**	0.257*	0.388**
E_Rigor	0.353**	0.325*	0.231	0.301*	0.246
E_Originality	0.25	0.09	0.205	0.112	0.315*
E_Significance	0.338**	0.217	0.354**	0.248	0.179
E_Conventions	0.193	0.004	0.19	0.017	0.325*

*p < 0.05; **p < 0.01; ***p < 0.001.

**Table 8:** Summary statistics of score agreement between experts and AI-supported collaborative marking ratings.

Measure	**Pearson r**	**Mean**	**SD**	***t*-value**	***p***	**95% Confidence interval** **(CI) for Indirect effect**	**Cohen’s *d***
Expert AI-supported collaborative marking total	0.415**	0.913	3.784	1.869	0.067	[−0.064,1.890]	0.241
Expert AI-supported collaborative marking rigor	0.325*	2.031	5.500	2.860	0.006**	[0.610,3.451]	0.369
Expert AI-supported collaborative marking originality	0.205	1.635	6.877	1.841	0.071	[−0.142,3.411]	0.238
Expert AI-supported collaborative marking significance	0.056	−1.088	5.404	−1.560	0.124	[−2.484,0.308]	−0.201
Expert AI-supported collaborative marking conventions	0.325*	1.167	4.491	2.012	0.049*	[0.006,2.327]	0.260

*p < 0.05; **p < 0.01; ***p < 0.001.

More specifically, the highest correlation was observed for the overall score (*r* = 0.415**), and the most significant difference appeared in the conventions dimension (*p* = 0.006). As shown in Figure 5B, most score differences fell within an acceptable margin. Compared to the AI without RAG, the 95% confidence interval of the mean difference was notably narrower, demonstrating improved consistency in the collaborative mode, which suggests enhanced metacognitive monitoring and reduced judgment variability during evaluation. These results indicate that AI-supported collaborative marking yields scoring outcomes that are more closely aligned with expert evaluations across most dimensions. Therefore, this collaboration approach partially reflects patterns of expert judgment and offers more reliable marking outcomes, especially in critical evaluation areas.

Within the AI-supported collaborative marking model, the overall scoring accuracy of student papers converges more closely to expert ratings than in evaluations without retrieval-augmented support. Specifically, after the Agent is grounded in an expert knowledge base generates the initial score, and students engage in questioning and other interactive activities with the Agent. This collaborative mechanism enables students to capitalize on the Agent’s knowledge resources and broaden their intellectual perspectives, while actively engaging in reflective comparison between their own judgments and expert-informed feedback. In particular, in the dimension of conventions, students’ in-depth exchanges with the Agent foster a more comprehensive understanding of academic standards and requirements, reflecting the internalization of explicit criteria and the strengthening of evaluative control. Moreover, in other key dimensions-including rigor, originality, significance, and conventions-students benefit from the interaction to varying extents, resulting in a systematic enhancement of accuracy across the evaluation rubric. This process ultimately provides a more precise and multidimensional perspective for academic marking, indicating that collaborative AI support functions not merely as a scoring aid but as a cognitive and metacognitive scaffold for evaluative reasoning development.

### 4.3 Progressing toward evaluative reflection in AI-supported collaborative marking

In response to RQ3, analysis of dialogue logs within the analyzed sample revealed that graduate students’ interaction strategies and behavioral patterns evolved during collaboration, shifting from fragmented trial-and-error approaches to more systematic and integrative evaluative practices. This behavioral shift may reflect core characteristics of self-regulated learning, as students demonstrate progressively strengthened capacities for planning evaluative strategies, monitoring AI-generated feedback, and reflecting on their scoring decisions. Problem-solving ability is a form of higher-order thinking, and the process of academic evaluation can be regarded as a complex problem-solving task. To closely examine students’ behavioral characteristics when conducting academic evaluation with the assistance of AI agents, we collected the dialogue logs between students and the expert-knowledge-based agent after the paper-assessing tasks were completed, thereby capturing the temporal patterns of students’ interaction behavior. This methodological choice was grounded in cognitive and educational psychology, as dialogic interaction data allow fine-grained observation of metacognitive monitoring, evaluative judgment formation, and regulatory decision-making processes unfolding over time.

**Figure 6:**
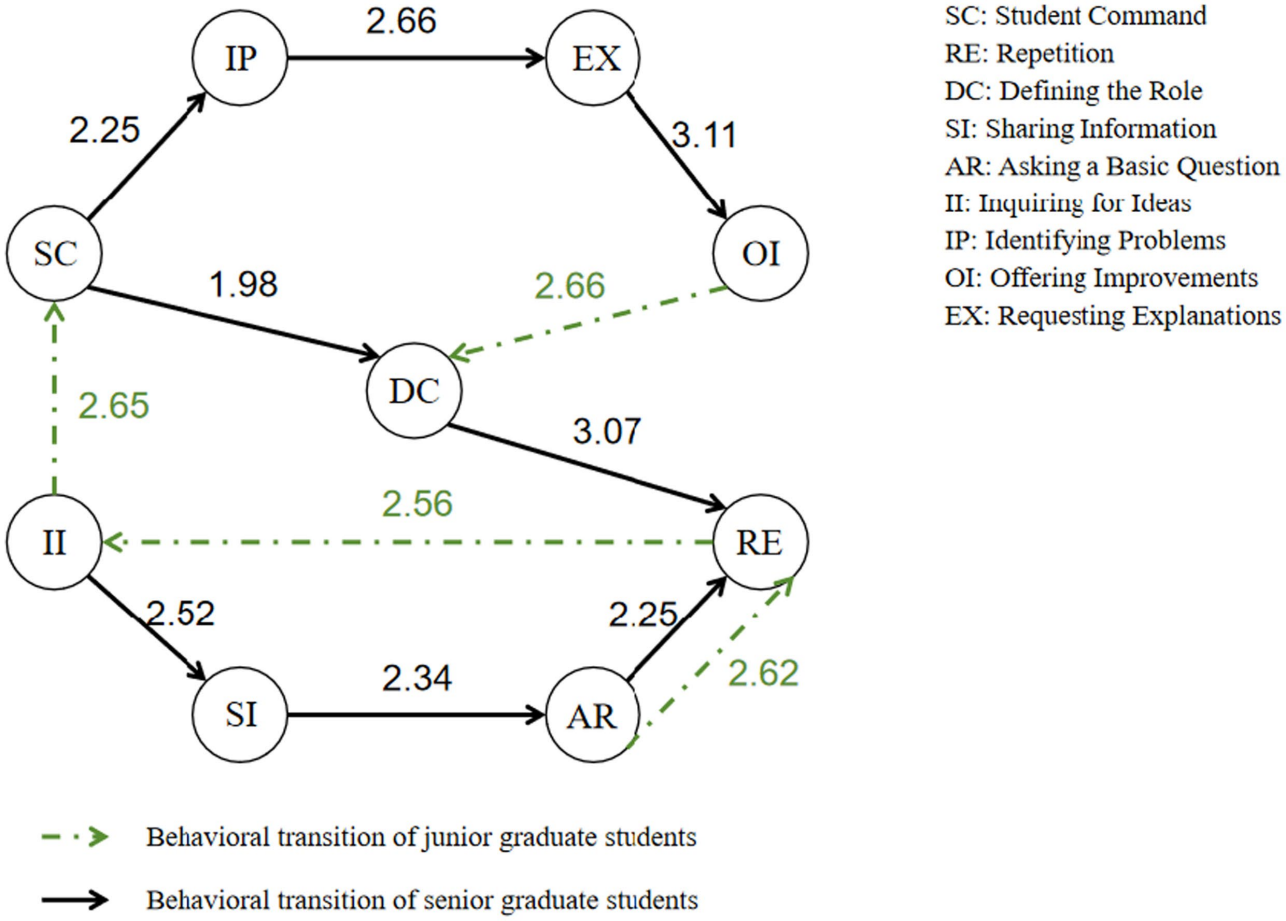
Behavioral transition patterns of different student types in the AI-supported collaborative marking.

Behavioral sequence analysis suggested clear distinctions in the interaction patterns between junior graduate students and more experienced senior graduate students. Figure 6 illustrate the flowchart distributions of these interaction strategies with the AI agent, highlighting critical behavioral transitions. In the figures, nodes represent distinct coded actions, links indicate significant behavioral connections, arrows denote the direction of transition, and the numbers on the links indicate the observed frequencies of transitions in the dataset.

On the one hand, junior graduate students showed a preference for behavioral sequences such as OI-DC and AR-RE-II. For example, a student might first identify the lack of specificity in the evaluation criteria and propose an improvement—"Could you include more detailed descriptions in the assessment of originality?” (OI). This corresponds to cognitive regulation and elaboration, indicating an attempt to compensate for an underdeveloped task representation by promoting higher quality information processing. Then proceed to define the AI’s role—"You are now acting as my academic assessment assistant. Please focus on evaluating originality and case analysis.” (DC), which reflects distributed metacognition. In this process, learners externalize metacognitive regulation and delegate part of the monitoring and judgment functions to the AI system (Pavlović, 2025). Another sequence involved initiating a basic inquiry—"Do you understand the importance of data literacy in modern education?” (AR), a typical information-seeking mechanism used to form an initial understanding of the task (Cheng et al., 2025)—followed by a repetition of the same question due to dissatisfaction with the response (RE), reflecting automation bias in which students attempt to test system reliability through repetition rather than deeper monitoring, and finally requesting ideas for evaluation—"Could you provide a perspective on how to evaluate this paper?” (II), a form of metacognitive control that reveals reliance on external strategic guidance when the task remains unclear. Notably, the AR-RE-II sequence indicates a progressive inquiry strategy, beginning with general questioning, followed by reinforcement through repetition, and concluding with seeking constructive suggestions, suggesting that their monitoring–calibration loop remains at an early stage and that they rely heavily on external scaffolding to compensate for limited internal regulatory ability.

On the other hand, senior graduate students exhibited more integrated behavioral clusters such as SC-IP-EX-OI, SC-DC-RE, and II-SI-AR-RE. For example, in the SC-IP-EX-OI sequence, students typically started by stating the evaluation task—"Please score this paper and explain your rationale.” (SC), which reflect planning and goal-setting processes and indicating more mature capacity for task framing (Heikkinen et al., 2024)—then identified shortcomings in the response and provided feedback (IP), an instance of counter-argumentation and reflective reasoning that reveals their ability to detect inconsistencies and revise system output (Vo et al., 2024; Onan et al., 2024)—followed by a detailed explanation request—"Please elaborate on the importance score and add supporting descriptions.” (EX), which represents metacognitive and conceptual regulation and helps learners better understand the inferential logic underlying evaluation criteria (Shields et al., 2024)—and concluded with a suggestion for improvement—"Can you include more content in the assessment of writing conventions?” (OI), further demonstrating cognitive regulation and elaboration to enhance the quality of the final judgment (Onan et al., 2024; Zheng et al., 2023). This pattern indicates that graduate students approached the evaluation task with a systematic and structured loop, following a “task clarification → problem identification → in-depth explanation → iterative refinement” model. In contrast, junior graduate students’ sequences like OI-DC and AR-RE-II reflected exploratory and trial-and-error patterns, lacking in systemic planning. Their behavior was more fragmented and featured higher repetition. Furthermore, they rarely engaged in Sharing Information (SI). SI reflects cognitive offloading, and students with more experience are more likely to provide background materials to reduce working memory load and enhance evaluative efficiency (Wahn et al., 2023). This difference may illustrate distinct approaches to regulating cognitive load across learner groups.

The observed differences in these behaviors can be attributed to multiple factors. Senior graduate students who have completed more than 1 year of graduate-level coursework typically demonstrate stronger technological literacy and richer experience in academic evaluation (Mansoor et al., 2024). Their interactions during the evaluation process tend to reflect more systematic and mature reasoning, aligning closely with the planning–monitoring–reflection cycle of SRL and indicating more stable metacognitive monitoring and strategy-adjustment mechanisms. In contrast, junior graduate students, lacking systematic academic preparation and limited exposure to technological tools, tend to exhibit weaker evaluative abilities and engage in more exploratory and trial-and-error behaviors. Their SRL cycle remains unstable, their monitoring is less developed, and they frequently rely on the system for task clarification and strategy supplementation. Moreover, these differences also highlight variations in students’ capacity to access and utilize academic resources, underscoring the need for personalized support in academic guidance. With structured guidance and feedback, students across different preparation levels can gradually strengthen their evaluative skills and narrow the performance gap.

## 5 Discussion

This study offers three novel contributions to the literature on AI-supported assessment. First, it demonstrates that AI-supported collaborative marking can improve both accuracy and inter-rater consistency compared with student-only or AI-only marking, particularly on originality and significance. Second, beyond performance outcomes, the findings suggest that AI-supported collaboration is associated with more systematic and reflective judgment strategies observed in students’ interaction behaviors and scoring practices. Third, the findings provide empirical evidence that graduate students at different stages of study exhibit distinct interaction patterns with AI, with senior students tend to demonstrate more structured cycles of planning, monitoring, and reflection, which may reflect developmental differences in self-regulated evaluative competence. Overall, these findings extend existing research by suggesting that AI-supported collaboration may provide a structured environment in which students engage with evaluation criteria and compare their judgments with benchmark standards. These findings are closely aligned with the study’s integrated theoretical framework. The observed shifts toward planning, monitoring, and reflection reflect core processes of self-regulated learning. The improved calibration to expert benchmarks supports evaluative judgment theory. In addition, the enhanced focus on higher-order reasoning under AI-supported conditions is consistent with cognitive load theory, as AI support may help structure the evaluation process and support reflective regulation during judgment activities while strengthening reflective regulation.

The AI-supported collaborative marking strategy proposed in this study shows potential for application in academic supervision. This research focuses on how AI can be used to compensate for students’ limitations in academic marking, explicitly framing collaborative marking as a psychological process of judgment calibration and standard internalization. The collaboration combines the advantages of generative AI in computation and information integration with students’ strengths in critical analysis and judgment, thereby enabling learners to externalize, compare, and reorganize their evaluative criteria during the marking process. As a result, the evaluation outcomes are not solely dependent on algorithmic outputs but are also subject to necessary human oversight, a configuration that aligns with psychological conceptions of evaluative judgment as a reflective and self-regulated cognitive activity. This complementary relationship enables the system to provide structured and comprehensive feedback while reducing potential ethical concerns, such as inappropriate inferences or harmful knowledge generation that may arise from AI use (Tamascelli et al., 2025). At the same time, careful attention should be given to ethical considerations in AI-supported evaluation environments, including potential risks such as automation bias, over-reliance on algorithmic feedback, and possible constraints on students’ independent evaluative reasoning. The results indicate that this mechanism is practically feasible in improving the accuracy and consistency of academic marking. When students participate as evaluators and compare their judgments with AI-generated evaluations, they gain opportunities to calibrate their own decisions, reflect on marking standards, and gradually internalize conventions of academic writing, a core mechanism through which evaluative judgment develops from externally guided comparison to internally regulated reasoning. The AI-supported collaborative marking model proposed in this study positions AI as a consistent evaluation partner, reducing subjectivity and inconsistency often present in peer review while retaining the educational value of fostering critical thinking and reflective learning. The consistency observed among students, AI, and AI-supported collaborative marking indicates that the collaborative process facilitated students’ understanding of evaluation criteria, suggesting enhanced alignment between learners’ internal judgment schemas and expert-referenced standards. By comparing their judgments with AI feedback aligned with expert standards, students refined their evaluative skills and developed a more systematic grasp of paper marking, reflecting strengthened metacognitive monitoring, error detection, and criterion awareness. Importantly, the collaborative marking framework should not only be regarded as a tool for improving scoring accuracy but also as a pedagogical strategy that integrates the training of research writing and evaluative skills, positioning assessment as an active learning process. By engaging students as active evaluators, the framework enables them to learn writing through the act of marking, thereby supporting a more comprehensive enhancement of academic competence.

With respect to dimension-level analysis, the study found that students performed better in evaluating rigor and originality, but demonstrated greater variability in assessing significance and conventions. This pattern reflects differences in cognitive abstraction and epistemic uncertainty across evaluation dimensions. This indicates that students were relatively adept at identifying logical structures and innovative aspects but less experienced in judging academic value, societal relevance, and adherence to writing and citation standards. The introduction of the AI Agent appears to help address these limitations by providing immediate feedback that aligned student evaluations more closely with expert standards. Through this process, AI feedback may help reduce uncertainty during evaluation and support the reorganization of evaluative attention across dimensions. In contrast to traditional unidirectional automated scoring, the AI-supported collaborative marking mechanism introduced here not only generates scores but also facilitates reciprocal feedback and learning, prompting students to actively reconcile discrepancies between their judgments and expert-aligned criteria. Students are able to understand the reasoning behind the scores and apply this knowledge to improve subsequent writing. Over time, such collaboration may facilitate students’ gradual internalization of expert standards and methods, thereby fostering more stable evaluative reasoning and sustained marking literacy.

In terms of data and model support, this study constructed a systematic dataset based on expert evaluation practices and academic supervision records to identify the challenges students encounter in authentic academic marking. The research team collected approximately 110,000 words of expert experience and supervision notes from 121 papers. These data made it possible to identify recurring cognitive difficulties in students’ evaluative processes, including incomplete task representations and inconsistent criterion application. The results indicate that relying solely on scores as a single indicator may obscure specific problems faced by students, whereas a collaborative mechanism that incorporates dimension-based analysis and feedback better aligns with the core objectives of academic supervision. Specifically, students performed relatively well in evaluating rigor and originality, demonstrating the ability to recognize logical structures and innovative aspects; however, they showed weaker performance in assessing significance and conventions, where difficulties emerged in evaluating academic value, societal relevance, and adherence to writing standards. A discrepancy that reflects uneven development of evaluative schemas across dimensions. Such differences contributed to deviations between student evaluations and expert benchmarks and suggest that focusing exclusively on score accuracy is insufficient (Xie et al., 2024). In this context, the integration of the Agent model can help address students’ shortcomings through feedback and provide judgments more closely aligned with expert standards, supporting cognitive stabilization and reducing extraneous evaluative load.

From the perspective of metacognitive and self-regulated learning development, the markedly different interaction patterns observed between novice and advanced students can be meaningfully interpreted (Geng and Su, 2025). Advanced students exhibited more systematized behavioral sequences (e.g., SC → IP → EX → OI), indicating more coherent internal models of evaluation tasks and more efficient metacognitive monitoring. They tended to articulate evaluative goals with greater clarity, identify and explain discrepancies, and iteratively optimize their strategies. This aligns with previous findings showing that higher-performing learners possess more refined task representations, more efficient metacognitive monitoring, and more consolidated domain knowledge structures (van der Graaf et al., 2022). In contrast, novice learners demonstrated exploratory and trial-and-error behavioral sequences (e.g., OI → DC or AR → RE → II), reflecting greater task-processing difficulty, unstable task representations, and restricted procedural regulation—characteristics typically associated with early stages of reflective judgment development (Sun et al., 2023; Wang et al., 2023). Their reliance on reactive rather than proactive monitoring helps explain their lower alignment with expert standards. Moreover, students’ behaviors of reliance, questioning, and revision collectively formed a metacognitively regulated dynamic loop of “trust–doubt–adjustment,” illustrating how AI feedback can activate monitoring, trigger reflective evaluation, and support judgment recalibration. When AI feedback aligned with students’ initial judgments, it contributed to a calibrated form of trust, which stabilized learners’ initial evaluative confidence and reduced uncertainty during early judgment formation. Divergence between the two activated metacognitive monitoring mechanisms, prompting learners to conduct reliability checks by re-querying, verifying, or requesting further explanation, thereby shifting students from intuitive judgment to deliberate evaluation. Throughout this process, students continuously compared AI feedback with their expectations and prior knowledge, enabling dynamic calibration of judgments (Jin et al., 2023). Behaviors such as correcting AI errors or requesting clarification of evaluative criteria directly reflected the activation of reflective reasoning and criteria clarification—core psychological mechanisms underlying evaluative judgment. Prior research indicates that students with stronger self-regulatory strategies are more likely to employ AI strategically during interaction, thereby achieving higher-quality learning outcomes (Jin et al., 2025). Furthermore, AI-supported learning processes are often integrated into cyclical structures of planning, iteration, and evaluation, fostering metacognitive monitoring and reflection throughout task engagement (Anders and Speltz, 2025). Consequently, this iterative calibration process offers a psychological explanation for the increased alignment between student scoring and expert standards. The AI agent also promoted reflective reasoning by explicitly presenting the rationale behind its evaluations. Its explanatory feedback foregrounded the logical relationships among factual claims, argument structures, and evidence quality, providing students with a stepwise evaluative pathway. Prior studies suggest that AI tools can effectively support all three phases of self-regulated learning—forethought, performance, and reflection—thereby strengthening metacognitive monitoring and regulation (Lan and Zhou, 2025). Empirical findings further show that students often evaluate, question, revise, or selectively adopt AI outputs, consistent with the mechanisms of criteria clarification and evidence verification in evaluative judgment (Liu et al., 2025). In the present study, such cues functioned as external metacognitive scaffolds that elicited self-questioning and cross-verification of evidence—critical components in the development of metacognitive monitoring and reflective judgment. By making discrepancies between students’ judgments and expert standards salient, AI feedback facilitated continual revision and structured reflection, thereby supporting the formation of more coherent evaluative pathways.

At present, the educational roles of artificial intelligence are mainly distributed across intelligent tutoring systems (ITSs), learner profiling, and predictive modeling (Chen X. et al., 2025). These applications guide students through personalized instruction and adaptive feedback, evaluate engagement and cognitive development, and provide real-time suggestions to support participation in higher-order thinking tasks (Stojanov et al., 2024; Liu et al., 2022; Wu et al., 2024). Other applications focus on language learning tasks, such as Grammarly (Khan et al., 2024), which offers real-time corrections and writing suggestions to improve grammatical accuracy and stylistic appropriateness. Such tools make it more convenient for students to strengthen language skills and facilitate the use of AI for writing improvement. However, compared with more complex AI-supported collaborative marking frameworks that address multidimensional aspects of academic writing—including rigor, originality, significance, and adherence to conventions—current explorations remain limited. Comprehensive evaluation of these dimensions relies heavily on the language understanding and logical reasoning capabilities of AI systems, as well as learners’ ability to interpret, monitor, and reconcile evaluative criteria across dimensions, which requires strategies different from traditional grammar-oriented marking. The AI-supported collaborative marking approach proposed in this study demonstrates adaptability to academic tasks involving extensive multidimensional data, and more importantly, illustrates how AI can be embedded within psychologically grounded assessment practices that support judgment formation and metacognitive regulation. In addition, our agent model and collaborative marking strategy effectively detect scores across specific dimensions and generate targeted revision suggestions, thereby supporting learners in identifying discrepancies, reflecting on criteria, and progressively stabilizing their evaluative reasoning. Current tools lack integration with expert knowledge bases and cannot provide multidimensional evaluation or actionable guidance, resulting in limited understanding of rigor, originality, significance, and conventions. By demonstrating how AI-mediated feedback can function as a cognitive–metacognitive scaffold within assessment activities, this study provides preliminary empirical support for psychological theories of assessment and learning, highlighting the role of guided comparison, reflective monitoring, and criterion internalization in the development of evaluative judgment. This study offers practical insights for designing dimension-specific academic guidance and AI-supported revision strategies.

## 6 Limitations

Although this study provides initial evidence for AI-supported collaborative marking, several limitations constrain the generalizability and ecological validity of the findings, particularly with respect to the construct validity of the psychological processes inferred in this research. First, the sample size and institutional context were relatively limited. The study focused exclusively on master’s students in educational technology, at a single university in China, whose disciplinary conventions and training backgrounds may influence external validity. The single-discipline focus and specific cultural environment further constrain generalizability. In addition, the behavioral interaction analysis was conducted on a relatively small subsample of participants, and therefore the interpretation of interaction patterns should be considered exploratory. Caution is therefore required when extending these findings to other academic domains or cross-cultural contexts or educational levels, and sweeping cross-disciplinary generalizations should be avoided. Second, the task design centered on structured academic essay writing and did not include more complex forms of academic production, such as theses, interdisciplinary projects, or extended research reports. These tasks typically involve cross-section integration, extended reasoning, and multilayered argumentation; thus, the applicability of the present findings to broader academic contexts requires further examination. In addition, the study primarily captured short-term interaction effects. Without a longitudinal or pretest-post test design, the findings only reflect immediate recalibration during AI-supported evaluation. The observed monitoring - reflection - adjustment cycles indicate that AI scaffolding can support short-term regulation of evaluative reasoning, but the design does not allow conclusions about whether these improvements persist over time. Accordingly, no conclusions can be drawn regarding the long-term development of students’ evaluative literacy or critical thinking abilities, and this temporal constraint should be taken into account when interpreting the findings. Regarding measurement and instrumentation, individual differences in AI-use experience, technological proficiency, and attitudes may have influenced participants’ interaction strategies and adoption behaviors. Moreover, core psychological constructs such as evaluative judgment, metacognitive monitoring, reflective reasoning, and cognitive load were inferred from observable behaviors, interaction patterns, and scoring outcomes rather than being directly measured using validated psychometric instruments, which limits the strength of causal claims about underlying cognitive mechanisms. Future research should incorporate standardized psychological measures and mixed-method designs to strengthen construct validity and clarify the mapping between observed behaviors and latent cognitive processes. In addition, both scoring rubrics and prompt templates may introduce structural biases, as rubrics embody disciplinary value judgments, and prompt framing shapes the form of AI-generated feedback. Although expert review and prompt standardization were used to mitigate these influences, sensitivity analyses of prompts and rubrics are recommended in subsequent studies. Furthermore, the task-based and time-bounded interactions within an experimental setting do not fully replicate the continuity and contextual richness of real instructional practice, such as semester-long thesis supervision or interdisciplinary research collaborations.

Finally, although the AI model employed in this study integrated expert knowledge bases and retrieval-augmented techniques, it still showed limitations in handling originality and complex reasoning, with some outputs diverging from expert judgment or displaying randomness. Additionally, the study relied on a single expert rater to provide benchmark evaluations for the full dataset of 121 papers. While this design helped maintain a consistent reference standard across all evaluation conditions, it may introduce potential bias and does not allow estimation of inter-rater reliability. Moreover, it should be noted that students evaluated their own work under certain conditions, which could have introduced self-enhancement bias, social desirability bias, and motivational distortion. To address this, we explicitly benchmarked all student evaluations against expert ratings and incorporated AI-assisted feedback to reduce potential subjective bias. These limitations delimit the extent to which AI feedback can be assumed to reliably support higher-order evaluative reasoning across all dimensions, suggesting that the psychological effects observed here should be interpreted in light of the current capabilities of AI systems.

## 7 Challenges and future work

Future research needs to further extend the Student-AI Agent collaborative assessment framework in several directions. To further establish the broad applicability of the proposed framework, future studies should validate it across a wider range of disciplinary contexts, multi-level student populations—such as undergraduates, master’s students, and doctoral candidates—and diverse educational settings. First, adaptability across disciplines and languages remains a major challenge. Different fields may interpret originality, significance, and conventions in divergent ways, and writing norms and scholarly expression vary in multilingual contexts. Therefore, large-scale and cross-cultural empirical studies are required to examine the generalizability and scalability of this framework. In this process, closer integration of domain expert knowledge bases and knowledge graphs deserves exploration, as these resources can provide stronger disciplinary contextual understanding and improve performance in recognizing originality and handling complex reasoning (Hu and Wang, 2024).

The continued expansion of educational scenarios and task genres is likewise essential. Although the present study focuses on graduate students and the structured genre of research paper writing, an AI-supported collaborative evaluation framework can be extended to undergraduate learners, K-12 students, and more complex academic products, including theses, interdisciplinary research projects, capstone designs, and extended academic reports. These tasks require learners to undertake higher-order conceptual integration, multi-step argumentation, and cross-domain reasoning, thereby providing a more comprehensive basis for examining the stability of the framework and the robustness of learners’ evaluative performance across developmental stages and writing genres (Krumsvik, 2024; Chen et al., 2022). Moreover, in the area of human–AI interaction, ensuring fairness and transparency while mitigating potential biases or inappropriate guidance from AI outputs will remain a critical issue in the design of collaborative systems (Chinta et al., 2024). Future research should incorporate additional safeguards at both methodological and system levels to mitigate potential threats to the validity of AI-generated output. Promising directions include involving multiple independent expert raters to improve benchmark reliability and reduce potential bias. Future work may also adopt ensemble or multi-model architectures to mitigate single-model effects. In addition, expanding expert knowledge bases through interdisciplinary corpora may improve the system’s ability to recognize originality and handle complex reasoning, while longitudinal assessments can further examine the stability of human-AI collaborative workflows over time.

Moreover, longitudinal research is crucial for elucidating the long-term educational effects of AI-supported collaborative evaluation. Existing studies largely emphasize short-term scoring accuracy and consistency, yet systematic tracking of learners’ development in critical thinking, evaluative judgment, and academic independence remains limited. Consequently, future work should employ longitudinal designs or delayed post-tests to determine whether the gains students acquire through sustained interaction with AI can be retained over time. Future studies should also expand the sample size used for behavioral interaction analysis to improve the robustness and generalizability of observed interaction patterns. Long-term tracking may include repeated measurements across multiple writing tasks, assessments of students’ ability to independently apply evaluative criteria without assistance, and analyses of temporal changes in the coherence, originality, and argumentative soundness of their reasoning processes. Accumulated cross-time data will facilitate a more systematic understanding of the sustained impact of AI-assisted evaluation on learners’ higher-order thinking and academic competencies, providing development-oriented empirical evidence for instructional practice.

Finally, future work should adopt systematic approaches to assess and enhance the fairness of AI-assisted collaborative evaluation. Key directions include integrating interpretability-enhanced interfaces that enable instructors and students to inspect the basis of AI-generated feedback; developing bias-detection dashboards to monitor systematic disparities across demographic or academic subgroups; and conducting fairness audits prior to deployment. Increasing the diversity of expert knowledge bases, testing the framework with multilingual and interdisciplinary datasets, and releasing ethically compliant mislabeled datasets will further strengthen transparency, reproducibility, and equitable learning outcomes. These efforts align with emerging research on fair and responsible AI in education and support the goal of ensuring that human-AI collaboration remains ethically grounded while maintaining instructional effectiveness.

Overall, future studies should not only focus on technical optimization but also address educational values and ethical considerations. Only through the combined advancement of cross-disciplinary collaboration, knowledge graph support, and interactional design can the AI-supported collaborative marking framework achieve the dual goals of educational equity and quality improvement. And future research should further investigate how sustained engagement with collaborative scoring can help students develop evaluative literacy, critical thinking, and independent research skills, positioning AI not merely as a scoring tool but as a genuine partner in the learning process.

## 8 Conclusion

This study introduces and validates a AI-supported collaborative marking strategy, which combines a structured collaborative framework with strategies specifically designed for academic supervision. Beyond proposing a technical framework, the study offers psychological insights into how evaluative judgment is constructed, calibrated, and refined through iterative comparison, feedback interpretation, and reflective adjustment, thereby linking AI-mediated interaction processes with theoretical mechanisms proposed in evaluative judgment and self-regulated learning research. The proposed framework is intended to analyze large volumes of academic marking data to identify students’ weaknesses and address them through an AI system grounded in an expert knowledge base. By integrating the generative capabilities of AI with students’ evaluative strengths, this collaborative marking approach differs from conventional AI-based methods. The framework is capable of evaluating manuscripts across multiple dimensions, including rigor, originality, significance, and adherence to academic conventions, while providing targeted recommendations for improvement. From a cognitive perspective, engagement with multidimensional criteria and expert-aligned feedback supports students in clarifying evaluation standards, monitoring discrepancies, and progressively internalizing norms of academic quality. Compared with marking conducted solely by students or AI, the collaborative approach aligns more closely with expert standards and delivers real-time feedback to guide students in identifying and developing areas for improvement. Importantly, the framework not only streamlines the academic evaluation process but also structures and elicits the core cognitive processes integral to evaluative judgment: metacognitive monitoring, criteria-based reasoning, and reflective revision, shifting the focus of marking from purely outcome-oriented to process- and education-oriented. The framework should not only be regarded as a step toward automation but also as a pedagogical tool, in which the AI-assisted grading process supports graduate students in learning academic writing. This study suggests that the AI-supported collaborative marking framework can provide multidimensional evaluation while also contributing to understanding of how students develop evaluative judgment through interaction with structured feedback and expert-informed reference points. This dual focus underscores its value as both an assessment mechanism and a developmental tool in academic supervision. This collaborative assessment framework demonstrates the potential of human–AI collaboration in educational evaluation, where AI functions as a learning partner that scaffolds reflective judgment, supports metacognitive regulation, and promotes the gradual formation of independent evaluative competence, and fosters reflective learning practices extending beyond single-course contexts. By embedding AI-supported marking within established psychological theories of assessment and learning, this study advances understanding of how academic competence, reflective judgment, and higher-order cognitive processes are cultivated in graduate education, thereby promoting a sustainable model of graduate education. At the same time, the findings should be interpreted with consideration of several methodological constraints discussed earlier, including the reliance on a single expert benchmark and the relatively small behavioral interaction subsample. Future research should therefore pursue longitudinal investigations, cross-institutional validation, and more direct measurement of relevant cognitive processes to further strengthen the empirical and theoretical foundations of AI-supported collaborative evaluation.

## Data Availability

The raw data supporting the conclusions of this article will be made available by the authors, without undue reservation.

## Footnotes

1
www.ref.ac.uk

## References

[ref1] Ajjawi Rola Tai Hong Meng Boud David Dawson Phillip. (2018). Developing Evaluative Judgement in Higher Education: Assessment for Knowing and Producing Quality Work London: Routledge.

[ref2] Alajami A. (2020). Beyond originality in scientific research: considering relations among originality, novelty, and ecological thinking. Think. Skills Creat. 38:100723. doi: 10.1016/j.tsc.2020.100723

[ref3] Alemdag E. Narciss S. (2025). Promoting formative self-assessment through peer assessment: peer work quality matters for writing performance and internal feedback generation. Int. J. Educ. Technol. High. Educ. 22:22. doi: 10.1186/s41239-025-00522-4

[ref4] Alsaiari O. Baghaei N. Lodge J. M. Noroozi O. Gašević D. Boden M. . (2026). Directive, metacognitive, or a blend of both? A comparison of AI-generated feedback types on student engagement, confidence, and outcomes. Comput. Educ. 10:100553. doi: 10.1016/j.caeai.2026.100553

[ref5] Anders A. D. Speltz E. D. (2025). Developing generative AI literacies through self-regulated learning: a human-centered approach. Comput. Educ. 9:100482. doi: 10.1016/j.caeai.2025.100482

[ref6] Anderson O. S. El Habbal N. Bridges D. (2020). A peer evaluation training results in high-quality feedback, as measured over time in nutritional sciences graduate students. Adv. Physiol. Educ. 44, 203–209. doi: 10.1152/advan.00114.2019, 32243221

[ref7] Bakeman R. Quera V. (2011). Sequential Analysis and Observational Methods for the Behavioral Sciences. Cambridge, UK: Cambridge University Press.

[ref8] Banihashem S. K. Bond M. Bergdahl N. Khosravi H. Noroozi O. (2025). A systematic mapping review at the intersection of artificial intelligence and self-regulated learning. Int. J. Educ. Technol. High. Educ. 22:50. doi: 10.1186/s41239-025-00548-8

[ref9] Barandas M. Folgado D. Santos R. Simão R. Gamboa H. (2022). Uncertainty-based rejection in machine learning: implications for model development and interpretability. Electronics 11:396. doi: 10.3390/electronics11030396

[ref10] Barkaoui K. (2011). Effects of marking method and rater experience on ESL essay scores and rater performance. Assess. Educ. Princ. Policy Pract. 18, 279–293. doi: 10.1080/0969594x.2010.526585

[ref11] Bearman M. Tai J. Dawson P. Boud D. Ajjawi R. (2024). Developing evaluative judgement for a time of generative artificial intelligence. Assess. Eval. High. Educ. 49, 893–905. doi: 10.1080/02602938.2024.2335321

[ref12] Boillos M. M. (2024). Peer review in early academic writing: impact vs. students’ beliefs. Innov. Lang. Learn. Teach. 18, 402–410. doi: 10.1080/17501229.2024.2311836

[ref13] Boud D. (2020). Challenges in reforming higher education assessment: a perspective from afar. Electron. J. Educ. Res. Eval. 26, 1–14. doi: 10.7203/relieve.26.1.17088

[ref14] Brimi H. M. (2011). Reliability of grading high school work in English. Pract. Assess. Res. Eval. 16:17. doi: 10.7275/j531-fz38

[ref15] Bui N. M. Barrot J. S. (2025). ChatGPT as an automated essay scoring tool in the writing classrooms: how it compares with human scoring. Educ. Inf. Technol. 30, 2041–2058. doi: 10.1007/s10639-024-12891-w

[ref16] Cano García E. Halbaut Bellowa L. Martins Gironelli L. Lluch Molins L. (2024). Online peer assessment in Galenic pharmacy: enhancing evaluative judgement in higher education. Med. Educ. Online 29:2409487. doi: 10.1080/10872981.2024.2409487, 39342639 PMC11441052

[ref17] Chan K. K. Y. Bond T. Yan Z. (2022). Application of an automated essay scoring engine to English writing assessment using many-facet Rasch measurement. Lang. Test. 40, 61–85. doi: 10.1177/02655322221076025

[ref18] Chen M. Cui Y. (2022). The effects of AWE and peer feedback on cohesion and coherence in continuation writing. J. Second. Lang. Writ. 57:100915. doi: 10.1016/j.jslw.2022.100915

[ref19] Chen J. Lai P. Chan A. Man V. Chan C.-H. (2022). AI-assisted enhancement of student presentation skills: challenges and opportunities. Sustainability 15:196. doi: 10.3390/su15010196

[ref20] Chen X. Xie H. Qin S. J. Wang F. L. Hou Y. (2025). Artificial intelligence-supported student engagement research: text mining and systematic analysis. Eur. J. Educ. 60:e70008. doi: 10.1111/ejed.70008

[ref21] Chen B. Zhang Z. Langrené N. Zhu S. (2025). Unleashing the potential of prompt engineering for large language models. Patterns 6:101260. doi: 10.1016/j.patter.2025.101260, 40575123 PMC12191768

[ref22] Cheng Y. Fan Y. Li X. Chen G. Gašević D. Swiecki Z. (2025). Asking generative artificial intelligence the right questions improves writing performance. Comput. Educ. Artif. Intell. 8:100374. doi: 10.1016/j.caeai.2025.100374

[ref23] Chinta S. V. Wang Z. Yin Z. Hoang N. Gonzalez M. Le Quy T. . (2024). FairAIED: navigating fairness, bias, and ethics in educational AI applications. Machine Learn. arXiv preprint, arXiv: 2407.18745. doi: 10.48550/arXiv.2407.18745

[ref24] Chong S. W. (2021). University students’ perceptions towards using exemplars dialogically to develop evaluative judgement: the case of a high-stakes language test. Asian Pac. J. Second Foreign Lang. Educ. 6:12. doi: 10.1186/s40862-021-00115-4

[ref25] Cicchetti D. V. (1994). Guidelines, criteria, and rules of thumb for evaluating normed and standardized assessment instruments in psychology. Psychol. Assess. 6:284.

[ref26] Cotton D. R. E. Cotton P. A. Shipway J. R. (2024). Chatting and cheating: ensuring academic integrity in the era of ChatGPT. Innov. Educ. Teach. Int. 61, 228–239. doi: 10.1080/14703297.2023.2190148

[ref27] Creswell J. W. Plano Clark V. L. (2011). Designing and Conducting Mixed Method Research. California, USA: Sage Publications.

[ref28] Crompton H. Burke D. (2023). Artificial intelligence in higher education: the state of the field. Int. J. Educ. Technol. High. Educ. 20:22. doi: 10.1186/s41239-023-00392-8

[ref29] de Bruin A. B. H. Roelle J. Carpenter S. K. Baars M. Efg M. R. E. (2020). Synthesizing cognitive load and self-regulation theory: a theoretical framework and research agenda. Educ. Psychol. Rev. 32, 903–915. doi: 10.1007/s10648-020-09576-4

[ref30] de Teixeira Melo A. Renault L. Caves L. S. D. Garnett P. Duarte Lopes P. Ribeiro R. . (2025). An AI tool for scaffolding complex thinking: challenges and solutions in developing an LLM prompt protocol suite. Cogn. Technol. Work 27, 651–693. doi: 10.1007/s10111-025-00817-6

[ref31] Dubois P. Lhotte R. (2023). Consistency and reproducibility of grades in higher education: a case study in deep learning [Epub ahead of print]. doi: 10.48550/arXiv.2305.07492

[ref32] Fischer J. Bearman M. Boud D. Tai J. (2024). How does assessment drive learning? A focus on students’ development of evaluative judgement. Assess. Eval. High. Educ. 49, 233–245. doi: 10.1080/02602938.2023.2206986

[ref33] Fisher D. P. Brotto G. Lim I. Southam C. (2025). The impact of timely formative feedback on university student motivation. Assess. Eval. High. Educ. 50, 622–631. doi: 10.1080/02602938.2025.2449891

[ref34] Funayama H. Sato T. Matsubayashi Y. Mizumoto T. Suzuki J. Inui K. (2022). Balancing cost and quality: an exploration of human-in-the-loop frameworks for automated short answer scoring. Comput. Lang. 13355, 465–476. doi: 10.1007/978-3-031-11644-5_38

[ref35] Geng X. Su Y.-S. (2025). The effects of different metacognitive patterns on students' self-regulated learning in blended learning. Comput. Educ. 227:105211. doi: 10.1016/j.compedu.2024.105211

[ref36] Giray L. (2023). Prompt engineering with ChatGPT: a guide for academic writers. Ann. Biomed. Eng. 51, 2629–2633. doi: 10.1007/s10439-023-03272-4, 37284994

[ref37] Goodman P. Robert R. C. Johnson J. E. (2020). Rigor in PhD dissertation research. Nurs. Forum 55, 611–620. doi: 10.1111/nuf.12477, 32515063

[ref38] Han C. Lu X. (2021). Interpreting quality assessment re-imagined: the synergy between human and machine scoring. Interpreting Soc. 1, 70–90. doi: 10.1177/27523810211033670

[ref39] Händel M. Harder B. Dresel M. (2020). Enhanced monitoring accuracy and test performance: incremental effects of judgment training over and above repeated testing. Learn. Instr. 65:101245. doi: 10.1016/j.learninstruc.2019.101245

[ref40] Heikkinen S. Cristea T. Saqr M. Malmberg J. Kleingeld A. Snijders C. . (2024). Sequence analysis and process mining perspectives to goal setting: what distinguishes business students with high and low self-efficacy beliefs? Smart Learn. Environ. 11:40. doi: 10.1186/s40561-024-00327-4

[ref41] Hesse F. Care E. Buder J. Sassenberg K. Griffin P. (2014). “A framework for teachable collaborative problem solving skills,” in Assessment and Teaching of 21st Century Skills: Methods and Approach, (Dordrecht, the Netherlands: Springer), 37–56.

[ref42] Hu Silan Wang Xiaoning. (2024). "FOKE: a personalized and explainable education framework integrating foundation models, knowledge graphs, and prompt engineering. [Epub ahead of print]

[ref43] Ibarra-Sáiz M. S. Gómez-Ruiz M. Á. Balderas A. Rodríguez-Gómez G. (2025). Improving learning through evaluative judgement and feedback using a technology-enhanced assessment environment. Technol. Knowl. Learn. 30:1–31. doi: 10.1007/s10758-025-09858-2

[ref44] Ilieva G. Yankova T. Ruseva M. Kabaivanov S. (2025). A framework for generative AI-driven assessment in higher education. Information 16:472. doi: 10.3390/info16060472

[ref45] Jin S.-H. Im K. Yoo M. Roll I. Seo K. (2023). Supporting students’ self-regulated learning in online learning using artificial intelligence applications. Int. J. Educ. Technol. High. Educ. 20:37. doi: 10.1186/s41239-023-00406-5

[ref46] Jin F. Lin C.-H. Lai C. (2025). Modeling AI-assisted writing: how self-regulated learning influences writing outcomes. Comput. Human Behav. 165:108538. doi: 10.1016/j.chb.2024.108538

[ref47] Jovic M. Papakonstantinidis S. Kirkpatrick R. (2025). From red ink to algorithms: investigating the use of large language models in academic writing feedback. Lang. Test. Asia 15:59. doi: 10.1186/s40468-025-00389-2

[ref48] Kasneci E. Sessler K. Küchemann S. Bannert M. Dementieva D. Fischer F. . (2023). ChatGPT for good? On opportunities and challenges of large language models for education. Learn. Individ. Differ. 103:102274. doi: 10.1016/j.lindif.2023.102274

[ref49] Ke Z. Ng V. (2019). Automated Essay Scoring: A Survey of the State of the Art. Palo Alto, California, USA: IJCAI.

[ref50] Khan M. O. Nazim M. Alzubi A. A. F. (2024). Exploring Arab EFL learners' attitudes: is Grammarly a game-changer in academic writing classes. Educ. Adm. Theory Pract. 30, 1365–3171. doi: 10.53555/kuey.v30i4.1612

[ref51] Knoth N. Tolzin A. Janson A. Leimeister J. M. (2024). AI literacy and its implications for prompt engineering strategies. Comput. Educ. Artif. Intell. 6:100225. doi: 10.1016/j.caeai.2024.100225

[ref52] Koch L. G. G. (1977). The measurement of observer agreement for categorical data. Biometrics 33, 159–174. doi: 10.2307/2529310 843571

[ref53] Koo T. K. Li M. Y. (2016). A guideline of selecting and reporting intraclass correlation coefficients for reliability research. J. Chiropr. Med. 15, 155–163. doi: 10.1016/j.jcm.2016.02.012, 27330520 PMC4913118

[ref54] Korzynski P. Mazurek G. Krzypkowska P. Kurasinski A. (2023). Artificial intelligence prompt engineering as a new digital competence: analysis of generative AI technologies such as ChatGPT. Entrep. Bus. Econ. Rev. 11, 25–37. doi: 10.15678/EBER.2023.110302

[ref55] Krumsvik R. J. (2024). Chatbots and academic writing for doctoral students. Educ. Inf. Technol. 30, 9427–9461. doi: 10.1007/s10639-024-13177-x

[ref56] Lan M. Zhou X. (2025). A qualitative systematic review on AI empowered self-regulated learning in higher education. NPJ Sci. Learn. 10:21. doi: 10.1038/s41539-025-00319-0, 40319057 PMC12049540

[ref57] Langer M. Baum K. Schlicker N. (2024). Effective human oversight of AI-based systems: a signal detection perspective on the detection of inaccurate and unfair outputs. Minds Mach. 35:1. doi: 10.1007/s11023-024-09701-0

[ref58] Langfeldt L. Nedeva M. Srlin S. Thomas D. A. (2020). Co-existing notions of research quality: a framework to study context-specific understandings of good research. Minerva 58, 115–137. doi: 10.1007/s11024-019-09385-2

[ref59] Lerchenfeldt S. Mi M. Eng M. (2019). The utilization of peer feedback during collaborative learning in undergraduate medical education: a systematic review. BMC Med. Educ. 19:321. doi: 10.1186/s12909-019-1755-z, 31443705 PMC6708197

[ref60] Levin I. Marom M. Kojukhov A. (2025). Rethinking AI in education: highlighting the metacognitive challenge. Broad Res. Artif. Intel. Neurosci. 16, 250–263. doi: 10.70594/brain/16.s1/21

[ref61] Li Y. Raković M. Srivastava N. Li X. Guan Q. Gašević D. . (2025). Can AI support human grading? Examining machine attention and confidence in short answer scoring. Comput. Educ. 228:105244. doi: 10.1016/j.compedu.2025.105244

[ref62] Li X. Yang H. Hu S. Geng J. Lin K. Li Y. (2022). Enhanced hybrid neural network for automated essay scoring. Expert. Syst. 39:e13068. doi: 10.1111/exsy.13068

[ref63] Ling G. Mollaun P. Xi X. (2014). A study on the impact of fatigue on human raters when scoring speaking responses. Lang. Test. 31, 479–499. doi: 10.1177/0265532214530699

[ref64] Liu S. Liu S. Liu Z. Peng X. Yang Z. (2022). Automated detection of emotional and cognitive engagement in MOOC discussions to predict learning achievement. Comput. Educ. 181:104461. doi: 10.1016/j.compedu.2022.104461

[ref65] Liu X. Xiao Y. Li D. (2025). Assessing strategic use of artificial intelligence in self-regulated learning: instrument development and evidence from Chinese university students. Int. J. Educ. Technol. High. Educ. 22:69. doi: 10.1186/s41239-025-00567-5

[ref66] Lu X. Mahesh A. Shen Z. Dudley M. Sano L. Wang X. (2025). Exploring LLM-generated feedback for economics essays: how teaching assistants evaluate and envision its use. Hum. Comput. Interact. 15878, 392–406. doi: 10.1007/978-3-031-98417-4_28

[ref67] Lu F.- I. Takahashi S. G. Kerr C. (2021). Myth or reality: self-assessment is central to effective curriculum in anatomical pathology graduate medical education. Acad. Pathol. 8:23742895211013528. doi: 10.1177/23742895211013528, 34027054 PMC8120525

[ref68] Mahshanian A. Shahnazari M. (2020). The effect of raters fatigue on scoring EFL writing tasks. Indones. J. Appl. Linguist. 10, 1–13. doi: 10.17509/ijal.v10i1.24956

[ref69] Mansoor H. M. H. Bawazir A. Alsabri M. A. Alharbi A. Okela A. H. (2024). Artificial intelligence literacy among university students—a comparative transnational survey. Front. Commun. 9:1478476. doi: 10.3389/fcomm.2024.1478476

[ref70] Marvin G. Hellen N. Jjingo D. Nakatumba-Nabende J. (2024). “Prompt engineering in large language models,” in Data Intelligence and Cognitive Informatics, eds. Jeena Jacob I. Piramuthu S. Falkowski-Gilski P. (Singapore: Springer Nature Singapore), 387–402.

[ref71] Mcconlogue Teresa (2020) Assessment and Feedback in Higher Education: a Guide for Teachers. London: UCL Press.

[ref72] Mchugh M. L. (2012). Interrater reliability: the kappa statistic. Biochem. Med. 22, 276–282. doi: 10.11613/BM.2012.031 PMC3900052 23092060

[ref73] Misgna H. On B.-W. Lee I. Choi G. S. (2024). A survey on deep learning-based automated essay scoring and feedback generation. Artif. Intell. Rev. 58:36. doi: 10.1007/s10462-024-11017-5

[ref74] Mohammed S. J. Khalid M. W. (2025). Under the world of AI-generated feedback on writing: mirroring motivation, foreign language peace of mind, trait emotional intelligence, and writing development. Lang. Test. Asia 15:7. doi: 10.1186/s40468-025-00343-2

[ref75] Morris W. Holmes L. Choi J. S. Crossley S. (2024). Automated scoring of constructed response items in math assessment using large language models. Int. J. Artif. Intell. Educ. 35, 559–586. doi: 10.1007/s40593-024-00418-w

[ref76] National Academies of Sciences, Engineering, and Medicine (2022). “7 item scoring,” in A Pragmatic Future for NAEP: Containing Costs and Updating Technologies, (Washington, DC: The National Academies Press).

[ref77] Ningrum S. Crosthwaite P. (2020). Gender-preferential language use in L1 and L2 argumentative essays? Evidence against lists of ‘gendered’ language features. Indones. J. Appl. Linguist. 10, 226–241. doi: 10.17509/ijal.v10i1.25100

[ref78] O’Connor B. P. (2000). Simple and flexible SAS and SPSS programs for analyzing lag-sequential categorical data. Behav. Res. Methods Instrum. Comput. 31, 718–726. doi: 10.3758/bf03200753, 10633992

[ref79] OECD (2026). OECD Digital Education Outlook 2026: Exploring Effective Uses of Generative AI in Education. Paris: OECD Publishing.

[ref80] Onan E. Biwer F. Abel R. Wiradhany W. de Bruin A. (2024). Optimizing self-organized study orders: combining refutations and metacognitive prompts improves the use of interleaved practice. NPJ Sci. Learn. 9:33. doi: 10.1038/s41539-024-00245-7, 38658711 PMC11043372

[ref81] Ortega-Ruipérez B. Correa-Gorospe J. M. (2024). Peer assessment to promote self-regulated learning with technology in higher education: systematic review for improving course design. Front. Educ. 9:1376505. doi: 10.3389/feduc.2024.1376505

[ref82] Ouyang F. Zheng L. Jiao P. (2022). Artificial intelligence in online higher education: a systematic review of empirical research from 2011 to 2020. Educ. Inf. Technol. 27, 7893–7925. doi: 10.1007/s10639-022-10925-9

[ref83] Pack A. Barrett A. Escalante J. (2024). Large language models and automated essay scoring of English language learner writing: insights into validity and reliability. Comput. Educ. Artif. Intell. 6:100234. doi: 10.1016/j.caeai.2024.100234

[ref84] Palermo C. (2022). Rater characteristics, response content, and scoring contexts: decomposing the determinates of scoring accuracy. Front. Psychol. 13:937097. doi: 10.3389/fpsyg.2022.937097, 36033049 PMC9399925

[ref85] Pavlović J. (2025). Constructivist psychology principles of human–AI collaboration. Front. Psychol. 16:1638774. doi: 10.3389/fpsyg.2025.1638774, 41415355 PMC12708894

[ref86] Raković M. Bernacki M. L. Greene J. A. Plumley R. D. Hogan K. A. Gates K. M. . (2022). Examining the critical role of evaluation and adaptation in self-regulated learning. Contemp. Educ. Psychol. 68:102027. doi: 10.1016/j.cedpsych.2021.102027

[ref87] Rivera H. (2022). Influence of citation practices on academic assessment. Cent. Asian J. Med. Hypotheses Ethics 3, 125–129. doi: 10.47316/cajmhe.2022.3.2.06

[ref88] Romeo G. Conti D. (2025). Exploring automation bias in human–AI collaboration: a review and implications for explainable AI. AI Soc. 41, 259–278. doi: 10.1007/s00146-025-02422-7

[ref89] Shields M. Calabro G. Selmeczy D. (2024). Active help-seeking and metacognition interact in supporting children’s retention of science facts. J. Exp. Child Psychol. 237:105772. doi: 10.1016/j.jecp.2023.105772, 37690348

[ref90] Starch D. Elliott E. C. (1912). Reliability of the grading of high-school work in English. Sch. Rev. 20, 442–457.

[ref91] Stojanov A. Liu Q. Koh J. H. L. (2024). University students’ self-reported reliance on ChatGPT for learning: a latent profile analysis. Comput. Educ. Artif. Intell. 6:100243. doi: 10.1016/j.caeai.2024.100243

[ref92] Sun J. C.-Y. Liu Y. Lin X. Hu X. (2023). Temporal learning analytics to explore traces of self-regulated learning behaviors and their associations with learning performance, cognitive load, and student engagement in an asynchronous online course. Front. Psychol. 13:1096337. doi: 10.3389/fpsyg.2022.1096337 36755979 PMC9901299

[ref93] Tamascelli Michael Bunch Olivia Fowler Blake Taeb Maryam Cohen Achraf (2025) "Academic advising Chatbot powered with AI agent. In Proceedings of the 2025 ACM Southeast Conference

[ref94] Taylor B. Kisby F. Reedy A. (2024). Rubrics in higher education: an exploration of undergraduate students’ understanding and perspectives. Assess. Eval. High. Educ. 49, 799–809. doi: 10.1080/02602938.2023.2299330

[ref95] Tomaszewski L. E. Zarestky J. Gonzalez E. (2020). Planning qualitative research: design and decision making for new researchers. Int J Qual Methods 19, 5–19. doi: 10.1177/1609406920967174

[ref96] Tomisu H. Ueda J. Yamanaka T. (2025). The cognitive mirror: a framework for AI-powered metacognition and self-regulated learning. Front. Educ. 10:1697554. doi: 10.3389/feduc.2025.1697554

[ref97] Topping K. J. Gehringer E. Khosravi H. Gudipati S. Jadhav K. Susarla S. (2025). Enhancing peer assessment with artificial intelligence. Int. J. Educ. Technol. High. Educ. 22:3. doi: 10.1186/s41239-024-00501-1

[ref98] Tsakeni M. Nwafor S. C. Mosia M. Egara F. O. (2025). Mapping the scaffolding of metacognition and learning by AI tools in STEM classrooms: a bibliometric–systematic review approach (2005–2025). J. Intelligence 13:148. doi: 10.3390/jintelligence13110148, 41295429 PMC12653222

[ref99] van der Graaf J. Lim L. Fan Y. Kilgour J. Moore J. Gašević D. . (2022). The dynamics between self-regulated learning and learning outcomes: an exploratory approach and implications. Metacogn. Learn. 17, 745–771. doi: 10.1007/s11409-022-09308-9

[ref100] Verma N. Getenet S. Dann C. Shaik T. (2025). Evaluating an artificial intelligence (AI) model designed for education to identify its accuracy: establishing the need for continuous AI model updates. Educ. Sci. 15:403. doi: 10.3390/educsci15040403

[ref101] Viera A. J. Garrett J. M. (2005). Understanding interobserver agreement: the kappa statistic. Fam. Med. 37, 360–363. doi: 10.1111/j.1365-2362.2005.01498.x, 15883903

[ref102] Vo K. Sarkar M. White P. J. Yuriev E. (2024). Metacognitive problem solving: exploration of students’ perspectives through the lens of multi-dimensional engagement. Chem. Educ. Res. Pract. 26, 141–157. doi: 10.1039/d4rp00096j

[ref103] Wahn B. Schmitz L. Gerster F. N. Weiss M. (2023). Offloading under cognitive load: humans are willing to offload parts of an attentionally demanding task to an algorithm. PLoS One 18:e0286102. doi: 10.1371/journal.pone.0286102, 37205658 PMC10198496

[ref104] Wang T. Li S. Tan C. Zhang J. Lajoie S. P. (2023). Cognitive load patterns affect temporal dynamics of self-regulated learning behaviors, metacognitive judgments, and learning achievements. Comput. Educ. 207:104924. doi: 10.1016/j.compedu.2023.104924

[ref105] Wei Y. Liu D. (2024). Incorporating peer feedback in academic writing: a systematic review of benefits and challenges. Front. Psychol. 15:1506725. doi: 10.3389/fpsyg.2024.1506725, 39660258 PMC11628301

[ref106] Wetzler E. L. Cassidy K. S. Jones M. J. Frazier C. R. Korbut N. A. Sims C. M. . (2024). Grading the graders: comparing generative AI and human assessment in essay evaluation. Teach. Psychol. 52, 298–304. doi: 10.1177/00986283241282696

[ref107] Wu T.-T. Lee H.-Y. Li P.-H. Huang C.-N. Huang Y.-M. (2024). Promoting self-regulation progress and knowledge construction in blended learning via ChatGPT-based learning aid. J. Educ. Comput. Res. 61, 1539–1567. doi: 10.1177/07356331231191125

[ref108] Wu L. Wang A. Dong Y. (2024). An empirical research on the development of pre-service Teachers'Human-machine collaborative instructional design abilities-Fromthe perspective of self-generated instruction theory (in Chinese). e-Educ. Res. 45, 105–112. doi: 10.13811/j.cnki.eer.2024.12.014

[ref109] Xi Z. Chen W. Guo X. He W. Ding Y. Hong B. . (2025). The rise and potential of large language model based agents: a survey. Sci. China Inf. Sci. 68:121101. doi: 10.1007/s11432-024-4222-0

[ref110] Xie X. (2024). Book review: developing evaluative judgement in higher education: assessment for knowing and producing quality work. Int. J. Educ. Liter. Stud. 12:304. doi: 10.7575/aiac.ijels.v.12n.1p.304

[ref111] Xie W. Jia P. Zhang G. Wang X. (2024). Are reviewer scores consistent with citations? Scientometrics 129, 4721–4740. doi: 10.1007/s11192-024-05103-2

[ref112] Xu X. Qiao L. Cheng N. Liu H. Zhao W. (2025). Enhancing self-regulated learning and learning experience in generative AI environments: the critical role of metacognitive support. Br. J. Educ. Technol. 56, 1842–1863. doi: 10.1111/bjet.13599

[ref113] Yan L. Pammer-Schindler V. Mills C. Nguyen A. Gašević D. (2025). Beyond efficiency: empirical insights on generative AI'S impact on cognition, metacognition and epistemic agency in learning. Br. J. Educ. Technol. 56, 1675–1685. doi: 10.1111/bjet.70000

[ref114] Yang L. Xin T. Zhang S. Yu Y. (2022). Predication of writing originality based on computational linguistics. J. Intelligence 10:124. doi: 10.3390/jintelligence10040124, 36547511 PMC9783314

[ref115] Yao G. Fan L. (2025). Cognitive load scale for AI-assisted L2 writing: scale development and validation. Front. Psychol. 16:1666974. doi: 10.3389/fpsyg.2025.1666974, 41245310 PMC12611650

[ref116] Yin S. Chen F. Chang H. (2022). Assessment as learning: how does peer assessment function in students' learning? Front. Psychol. 13:912568. doi: 10.3389/fpsyg.2022.912568, 35832911 PMC9271947

[ref117] Zhai C. Wibowo S. Li L. D. (2024). The effects of over-reliance on AI dialogue systems on students' cognitive abilities: a systematic review. Smart Learn. Environ. 11:28. doi: 10.1186/s40561-024-00316-7

[ref118] Zheng L. Long M. Chen B. Fan Y. (2023). Promoting knowledge elaboration, socially shared regulation, and group performance in collaborative learning: an automated assessment and feedback approach based on knowledge graphs. Int. J. Educ. Technol. High. Educ. 20:46. doi: 10.1186/s41239-023-00415-4

[ref119] Zimmerman B. Schunk D. (2011). “Self-regulated learning and performance: an introduction and an overview,” in Handbook of Self- Regulation of Learning and Performance, (New York: Routledge), 1–15.

[ref120] Zixin C. Wang J. Li Y. Li H. Shi C. Zhang R. . (2025). CoGrader: transforming instructors' assessment of project reports through collaborative LLM integration. Hum. Comput. Interact. 1–18. doi: 10.1145/3746059.3747670

[ref121] Zong Z. Schunn C. D. Wang Y. (2020). Learning to improve the quality peer feedback through experience with peer feedback. Assess. Eval. High. Educ. 46, 973–992. doi: 10.1080/02602938.2020.1833179

